# Statin use and risk of colorectal cancer in patients with inflammatory bowel disease

**DOI:** 10.1016/j.eclinm.2023.102182

**Published:** 2023-08-24

**Authors:** Jiangwei Sun, Jonas Halfvarson, David Bergman, Fahim Ebrahimi, Bjorn Roelstraete, Paul Lochhead, Mingyang Song, Ola Olén, Jonas F. Ludvigsson

**Affiliations:** a Department of Medical Epidemiology and Biostatistics, Karolinska Institutet, Stockholm, Sweden; b Department of Gastroenterology, Faculty of Medicine and Health, Örebro University, Örebro, Sweden; c Department of Gastroenterology and Hepatology, Clarunis - University Center for Gastrointestinal and Liver Diseases, Basel, Switzerland; d Medicines Research Centre, GSK, Stevenage, UK; e Departments of Epidemiology and Nutrition, Harvard T.H. Chan School of Public Health, Harvard University, Boston, MA, USA; f Clinical and Translational Epidemiology Unit and Division of Gastroenterology, Massachusetts General Hospital and Harvard Medical School, Boston, MA, USA; g Clinical Epidemiology Division, Department of Medicine Solna, Karolinska Institutet, Stockholm, Sweden; h Sachs’ Children and Youth Hospital, Stockholm South General Hospital, Stockholm, Sweden; i Department of Clinical Science and Education Södersjukhuset, Karolinska Institutet, Stockholm, Sweden; j Department of Pediatrics, Örebro University Hospital, Örebro, Sweden; k Division of Digestive and Liver Disease, Department of Medicine, Columbia University Medical Center, New York, NY, USA

**Keywords:** Inflammatory bowel disease, Statin, Colorectal cancer, Cohort

## Abstract

**Background:**

Statin use has been linked to a reduced risk of advanced colorectal adenomas, but its association with colorectal cancer (CRC) in patients with inflammatory bowel disease (IBD) - a high risk population for CRC - remains inconclusive.

**Methods:**

From a nationwide IBD cohort in Sweden, we identified 5273 statin users and 5273 non-statin users (1:1 propensity score matching) from July 2006 to December 2018. Statin use was defined as the first filled prescription for ≥30 cumulative defined daily doses and followed until December 2019. Primary outcome was incident CRC. Secondary outcomes were CRC-related mortality and all-cause mortality. Cox regression estimated adjusted hazard ratios (aHRs) and 95% confidence intervals (CIs).

**Findings:**

During a median follow-up of 5.6 years, 70 statin users (incidence rate (IR): 21.2 per 10,000 person-years) versus 90 non-statin users (IR: 29.2) were diagnosed with incident CRC (rate difference (RD), −8.0 (95% CIs: −15.8 to −0.2 per 10,000 person-years); aHR = 0.76 (95% CIs: 0.61 to 0.96)). The benefit for incident CRC was duration-dependent in a nested case-control design: as compared to short-term use (30 days to <1 year), the adjusted odd ratios were 0.59 (0.25 to 1.43) for 1 to <2 years of use, 0.46 (0.21 to 0.98) for 2 to <5 years of use, and 0.38 (0.16 to 0.86) for ≥5 years of use (*P*_for tread_ = 0.016). Compared with non-statin users, statin users also had a decreased risk for CRC-related mortality (IR: 6.0 vs. 11.9; RD, −5.9 (−10.5 to −1.2); aHR, 0.56 (0.37 to 0.83)) and all-cause mortality (IR: 156.4 vs. 231.4; RD, −75.0 (−96.6 to −53.4); aHR, 0.63 (0.57 to 0.69)).

**Interpretation:**

Statin use was associated with a lower risk of incident CRC, CRC-related mortality, and all-cause mortality. The benefit for incident CRC was duration-dependent, with a significantly lower risk after ≥2 years of statin use.

**Funding:**

This research was supported by 10.13039/501100006636Forte (i.e., the 10.13039/501100006636Swedish Research Council for Health, Working Life and Welfare).

**Research in context**

**Evidence before this study**

We searched PubMed and Web of Science for studies on the association between statin use and colorectal cancer (CRC) in patients with inflammatory bowel disease (IBD), from database inception to April 30, 2023, with a combination of the following keywords ((inflammatory bowel disease OR IBD OR Crohn's disease OR ulcerative colitis) AND (statin OR hydroxymethylglutaryl coenzyme A reductase inhibitors) AND (colorectal cancer OR CRC OR colorectal neoplasm) AND (observational study OR randomized controlled trial)) and no language restriction. Overall, only a few observational studies had investigated the association between statin use and risk of CRC in IBD patients, but with substantial between-study heterogeneity and inconclusive results, suffering from small sample size, short follow-up time, and different biases. Possibly due to extended study duration required and low incidence of CRC, no RCT exists to date to investigate statins as chemopreventive agents against CRC in IBD patients.

**Added value of this study**

In this nationwide, propensity score matched cohort study in Sweden, statin use was associated with a lower risk of incident CRC, CRC-related mortality, and all-cause mortality in the biopsy-confirmed IBD patients. The number needed to treat to avoid one incident CRC, CRC-related death, and any death within 10 years after statin initiation was 227, 200, and 21, respectively. The benefits tended to be duration-dependent, with a significantly lower risk after more than two years of use. The benefits for incident CRC and CRC-related mortality were mainly observed in patients with UC, in patients diagnosed with IBD at age <50 years, in patients with longer IBD duration, for CRC-related mortality in colon cancer, and in early-stage CRC.

**Implications of all the available evidence**

While further research is required to validate our findings, our data suggest that statins may lower the risk of CRC in patients with IBD.

## Introduction

Inflammatory bowel disease (IBD) is a chronic gastrointestinal (GI) disease with a relapsing-remitting manner, encompassing Crohn’s disease (CD), ulcerative colitis (UC), and IBD-unclassified (IBD-U).^1^ Although incidence of IBD has begun to stabilize or even decrease in some western countries,^2^ its economic and social burden remains high due to high prevalence, young peak age of onset, and life-long therapy.^3^ Patients with IBD are at an approximately 1.5-fold increased risk of CRC,^4^^,^^5^ but estimates vary by disease duration, age at diagnosis, and extent of inflammation.^6^

Assuming that the majority of CRC in IBD patients occur as a consequence of chronic inflammation,^7^ control of mucosal inflammation may potentially prevent CRC development.^8^ For decades, chemoprevention agents that would reduce or even prevent inflammation-associated carcinogenesis have been the topic of investigation. To date, administration of 5-aminosalicylate (5-ASA, a drug with nonspecific anti-inflammatory effect) to patients with UC as a means of chemoprevention is advocated by most contemporary guidelines.^9^^,^^10^

Statins are primarily prescribed for treating hypercholesterolemia and preventing cardiac morbidity and mortality.^11^ In addition to the lipid-lowering effect, evidence from in vitro and animal studies have also reported anti-inflammatory, anti-proliferative, pro-apoptotic, and anti-neoplastic effects of statins.^11^ Our previous findings suggested statin use was associated with a lower risk of CD.^12^ However, evidence from human studies for the potential chemopreventive effect of statin on CRC remains inconclusive.

Since 2010, several meta-analyses and systematic reviews have reported a significantly protective effect of statin on CRC risk among non-IBD patients,^13^, ^14^, ^15^, ^16^ but this benefit is only observed in observational studies, not in randomized controlled trials (RCTs). Several observational studies have investigated the association between statin use and risk of CRC in IBD patients, with substantial between-study heterogeneity and inconclusive results^17^, ^18^, ^19^, ^20^ (summarized in Supplementary Table S1). These studies have suffered from various limitations (e.g., small sample size,^17^^,^^18^ restriction to single centers,^17^, ^18^, ^19^ inclusion of prevalent CRC cases,^18^ varied statin assessment (e.g., electronic prescriptions^18^ vs. self-reported^20^)). Moreover, possibly due to extended study duration required and fairly low incidence of CRC,^7^ no RCT exists to date to investigate statins as chemopreventive agents against CRC in IBD patients. As a result, a well-designed observational study that overcomes limitations of earlier studies is likely to provide the valuable evidence on this topic.

We therefore conducted a propensity-score matched cohort study to investigate the association between statin use and risk of CRC, CRC-related mortality, and all-cause mortality in patients with biopsy-confirmed IBD using a new user design.

## Methods

### Data sources

This cohort study was based on the nationwide histopathology cohort, Epidemiology Strengthened by histoPathology Reports in Sweden (ESPRESSO),^21^ and several healthcare registers in Sweden (e.g., the Total Population Register, the Prescribed Drug Register, the National Patient Register, the Cancer Register, the Cause of Death Register, and the Swedish Longitudinal Integrated Database for Health Insurance and Labour Market Studies, see eMethods for details), which were cross-linked by the unique personal identity number assigned to all residents.^22^ The prespecified analysis plan is presented in the Supplementary Material.

### Ethics

This study was approved by the Stockholm Ethics Review Board (2014/1287-31/4, 2018/972-32, and 2022-05774-02). Individual informed consent was waived as the study was register-based.^23^

### Individuals with IBD

IBD patients were identified as those with ≥1 biopsy indicating IBD in the ESPRESSO and ≥1 International Classification of Disease (ICD) code for IBD in the NPR (see Supplementary Table S2 for the definition of IBD subtypes).^2^ Such diagnostic approach has been validated with a positive predictive value (PPV) of 95%.^24^ To minimize immortal time bias, date of IBD diagnosis was defined as the latter date of the first ICD or first biopsy code.

### Statin exposure

**Fig. 1:**
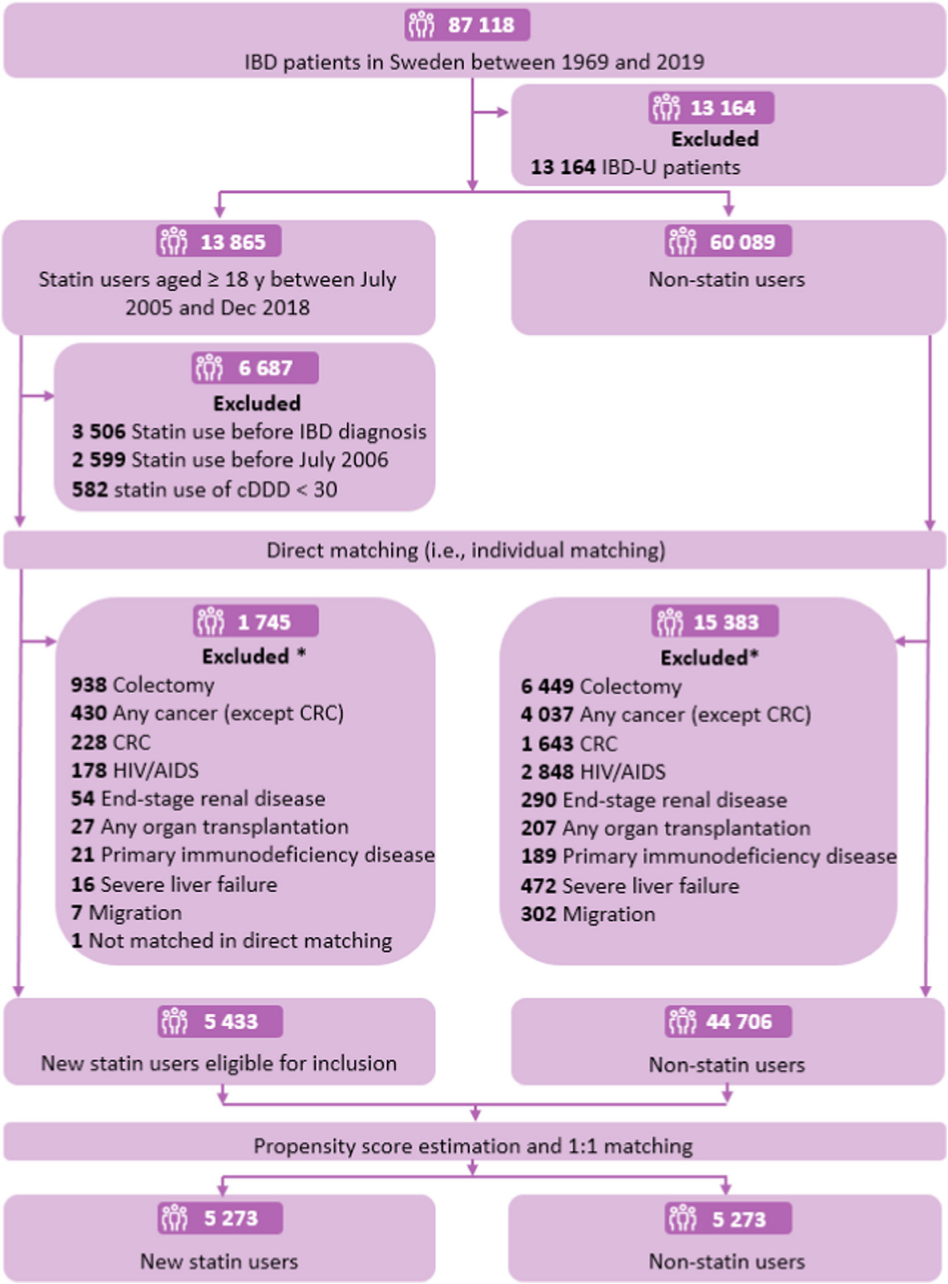
Flowchart of patient inclusion in the study cohort of statin use with risk of colorectal cancer (CRC) in patients with inflammatory bowel disease (IBD). HIV/AIDS: human immunodeficiency virus/acquired immunodeficiency syndrome; IBD-U: IBD-unclassified; ∗: some patients may meet multiple exclusion criteria.

Statin use was identified from the Prescribed Drug Register by the Anatomical Therapeutic Chemical (ATC) classification system code C10AA, including lipophilic statins (simvastatin and atorvastatin) and hydrophilic statins (pravastatin and rosuvastatin) (see Supplementary Table S3 for ATC codes). Statin users were identified as those aged ≥18 years and with a statin prescription for ≥30 cumulative defined daily doses (DDD, a standardized measure of average daily drug consumption by the World Health Organization) (cDDD) from July, 2006 or later (consistent with prior studies^25^^,^^26^). The date that an individual first attained a cDDD ≥30 for a statin was defined as the index date for statin exposure. To ensure a new-user design,^27^ individuals who had any statin prescription before July 2006, before IBD diagnosis or before the first attainment of cDDD ≥30 were excluded (Fig. 1). In an intention-to-treat analysis, all individuals were analyzed based on the groups to which they were initially allocated.

### Matching

We applied a two-step matching process to establish the cohort. In the direct matching step, statin users and non-statin users were individually matched on age at IBD diagnosis (<18, 18–<40, 40–<60, and ≥60), sex, IBD subtype (CD or UC), and calendar year at IBD diagnosis (1969–1989, 1990–1999, 2000–2009, 2010–2018). IBD-U patients were not considered duo to diagnostic uncertainty. To ensure similar opportunities for care and treatment,^25^ non-statin users should have a random drug prescription (except IBD medications, see Supplementary Table S3 for ATC codes) within three months before or after the start date of statin treatment in the matched statin users. All identified non-statin users that were matched to a given statin user were assigned the same index date as the index statin user. We excluded individuals who had a record of migration, received a colectomy, or had a diagnosis of any of the following diseases before the index date: cancers, human immunodeficiency virus/acquired immunodeficiency syndrome, primary immunodeficiency disease, end-stage renal disease, severe liver failure, and any organ transplantation (see Supplementary Table S3 for procedure and ICD codes, Fig. 1).

In the propensity score matching step (performed in the PSMATCH procedure in SAS version 9.4), statin users and non-statin users were matched in a 1:1 ratio by using a greedy nearest-neighbor matching algorithm without replacement, with a caliper of 0.2 standard deviation of the logit of the propensity score.^28^ The propensity score that predicted probability of statin use was derived from a logistic regression model that was adjusted for demographic and socioeconomic characteristics, comorbidities, and co-medications identified up to and including the index date to minimize potential confounding (see eMethods for details). Standardized mean difference was used to examine the balance of a covariate and imbalance was defined as a standardized difference value greater than 0.1.

### Follow-up and outcome ascertainment

Follow-up began at index date until the first recorded date of incident CRC, proctocolectomy, emigration, death, or end of follow-up on December 31, 2019. A patient who had a colectomy but had an intact rectum was considered at risk of rectal cancer until proctectomy (see Supplementary Table S3 for procedure codes). The primary outcome was incident CRC (see Supplementary Table S3 for ICD codes), ascertained from the Cancer Register. Secondary outcomes were CRC-related mortality and all-cause mortality (see Supplementary Table S3 for ICD codes), ascertained from the Cause of Death Register.

### Statistics and include all the statistical analysis

We chose the intention-to-treat analysis as the main analysis. Incidence rates and their differences with 95% confidence intervals (CIs) for incident CRC, CRC-related mortality, and all-cause mortality were calculated using Poisson regression. To account for competing risk, we applied a subdistribution hazard model to estimate hazard ratios (HRs) and 95% CIs, using time since index date as the underlying time scale and stratified by the matched pair. Competing events included proctocolectomy, emigration, and death for analyses of incident CRC; death from other causes or emigration for analyses of CRC-related mortality; and emigration for analyses of all-cause mortality. Cumulative incidence function in the stratified subdistribution hazard model was applied to estimate the cumulative incidence of the primary and secondary outcomes over follow-up period.

We reported the number needed to treat (NNT, estimated as the reciprocal of the cumulative incidence difference)^29^ to avoid one event at 10-year since it is the most commonly used time period for CRC.^30^ In our study, 18.8% of participants had a follow-up >10 years.

### Secondary analyses

Three secondary analyses were conducted. First, to assess the relationship between continuous statin treatment and outcomes, we conducted an as-treated analysis where we further censored the follow-up at the end of the first treatment episode, in addition to the abovementioned censoring criteria. We constructed the treatment episode by assigning a duration to each prescription corresponding to the number of DDDs dispensed. Second, to evaluate whether cumulative duration of statin use was associated with the outcomes, we additionally conducted a nested case-control study in statin users. Using the method of incidence density sampling, for each case exposed to statin and with later studied outcomes, we randomly selected up to five statin users who did not develop the interested outcomes (controls). They were individually matched on age at index date, sex, IBD subtype, IBD duration, and duration of follow-up. We applied the conditional logistic regression to estimate odds ratios (ORs) with 95% CIs. The cumulative duration of statin use was assessed between first prescription and end of follow-up, and categorized into predefined categories (30 days–<1 year (reference group), 1–<2 years, 2–<5 years, and ≥5 years cDDD). Trends in the dose-response association were tested by using the duration strata as an independent variable in the model. Third, we repeated the main analysis without accounting for competing risks.

### Subgroup analyses and sensitivity analyses

We assessed whether the associations varied by pre-specified factors, including age at the index date (<60 or ≥60 years), sex, calendar period for IBD diagnosis (1969–1999 or 2000–2018), educational attainment (0–9, 10–12, ≥13 years, or “missing”), IBD subtype (CD or UC), age at IBD diagnosis (<50 or ≥50 years), duration of IBD (<10 or ≥10 years), type of statins initially treated (lipophilic statins: simvastatin or atorvastatin), type of CRC (colon cancer or rectal cancer), location of colon cancer (left-sided or right-sided, see Supplementary Table S3 for the definition), and cancer stage (measured by tumour node metastasis (TNM) classification^31^: I–II or III–IV). Hydrophilic statins was not investigated due to small sample size.

Several sensitivity analyses were conducted to test robustness of our results. First, to reduce the influence by misclassification of covariates, we constructed a model in which covariates were adjusted as time-varying variables. Second, to reduce potential influence by comorbidities, we excluded individuals with a history of CVD or diabetes before statin initiation. Third, to minimize bias due to detection bias, surveillance bias, or reverse causation, we discarded the first year and the first two years of follow-up from the analysis. Fourth, we evaluated the potential influence from unmeasured confounding by calculating E-values.^32^ Finally, we performed a negative control analysis using fracture as a negative control outcome to assess potential biases in our study.^33^

Data analyses were performed using SAS version 9.4 (SAS Institute Inc, Cary, NC), Stata (version 16.1; StataCorp LP, College Station, TX), and R version 3.6.0 (packages “forestplot” and “ggplot2” for visualizing Fig. 3). A two-sided *P* ≤ 0.05 was considered statistically significant.

### Role of the funding source

The funder of the study had no role in study design, data collection, data analysis, data interpretation, or writing of the report.

## Results

**Fig. 2:**
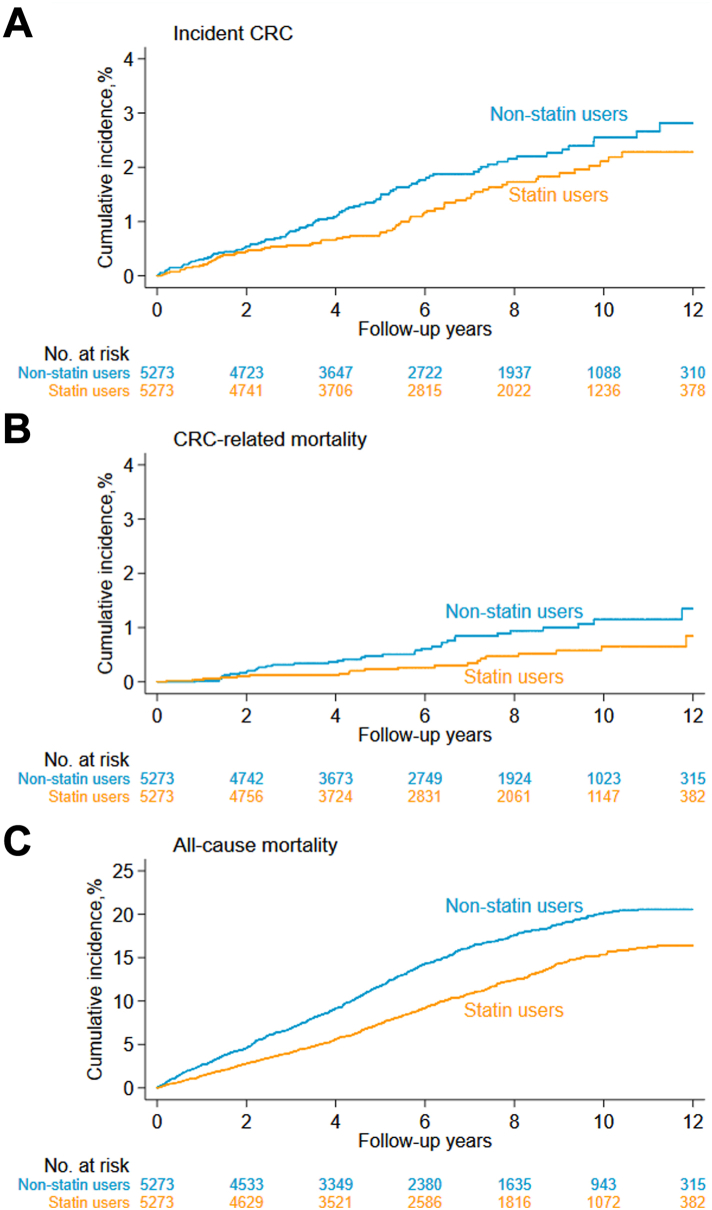
Cumulative incidence of incident colorectal cancer (CRC), CRC-related mortality, and all-cause mortality among statin-users and non-statin users. The *P* values for the three comparisons were 0.019, 0.0040, and <0.0001, respectively, and were derived from the stratified subdistribution hazard model.

In total, we identified 5433 new statin users and 44,706 non-statin users after direct matching (Fig. 1). Statin users tended to be older (mean age at IBD diagnosis: 51.6 vs. 39.6 years), more males (57.2% vs. 48.7%), and more likely to receive an IBD diagnosis before 2000 (Supplementary Table S4). After 1:1 propensity score matching, the final cohort included 5273 pairs of statin users and nonusers (Table 1). All standardized mean differences were <0.1 between the two groups, indicating good covariate balance. Mean age at IBD diagnosis was 51.7 years, while mean age at index date was 62.6 years. The majority of individuals were diagnosed with UC (69.9%) and were male (56.9%). During a median follow-up of 5.6 years (interquartile range: 3.0 to 9.0 years), we observed 160 incident CRC cases (users: 70, non-statin users: 90), 57 CRC-related deaths (users: 20, non-statin users: 37), and 1239 deaths (users: 520, non-statin users: 719). Compared to non-statin users, statin users had a lower cumulative incidence of the primary and secondary outcomes (Fig. 2). The 10-year cumulative incidence differences of incident CRC, CRC-related mortality, and all-cause mortality were 0.44%, 0.50%, and 4.82%, respectively. Therefore, the NNT to avoid one incident CRC, CRC-related death, and any death within 10 years after statin initiation was 227, 200, and 21, respectively.

**Table 1:** Characteristics of statin users and non-statin users before and after propensity score (PS) matching.

Characteristic	Statin users (n = 5273)	Non-statin users (n = 5273)	Standardized difference	
			Before PS matching	After PS matching
Age at IBD diagnosis, years			0.351	−0.007
Mean ± SD	51.7 ± 13.3	51.8 ± 15.2		
Median (IQR)	52.0 (42.6–60.8)	51.4 (41.9–61.1)		
Categories 1, n (%)				
<18	20 (0.4)	20 (0.4)	0	0
18–<40	1004 (19.0)	1004 (19.0)	0	0
40–<60	2822 (53.5)	2822 (53.5)	0	0
≥60	1427 (27.1)	1427 (27.1)	0	0
Categories 2, n (%)				
<50	2349 (44.5)	2416 (45.8)		
≥50	2924 (55.5)	2857 (54.2)		
Sex, n (%)				
Male	3000 (56.9)	3000 (56.9)	0	0
Female	2273 (43.1)	2273 (43.1)	0	0
Calendar period at IBD diagnosis, n (%)				
1969–1989	359 (6.8)	359 (6.8)	0	0
1990–1999	1384 (26.3)	1384 (26.3)	0	0
2000–2009	2771 (52.6)	2771 (52.6)	0	0
2010–2018	759 (14.4)	759 (14.4)	0	0
IBD subtype, n (%)				
CD	1589 (30.1)	1589 (30.1)	0	0
UC	3684 (69.9)	3684 (69.9)	0	0
Country of birth, n (%)			−0.016	0.004
Nordic country	4870 (92.4)	4865 (92.3)		
Others	403 (7.6)	408 (7.7)		
Educational attainment, n (%)				
0–9	1417 (26.9)	1465 (27.8)	0.152	−0.021
10–12	2313 (43.9)	2283 (43.3)	−0.067	0.011
≥13	1147 (21.8)	1061 (20.1)	−0.197	0.037
Missing	396 (7.5)	464 (8.8)	0.247	−0.059
Duration of IBD, years			0.217	−0.028
Mean ± SD	10.9 ± 7.4	11.1 ± 7.5		
Median (IQR)	9.9 (5.1–15.5)	10.1 (5.3–15.6)		
Categories, no. (%)				
<10	2676 (50.8)	2621 (49.7)		
≥10	2597 (49.3)	2652 (50.3)		
Age at index date, years^a^			0.500	−0.023
Mean ± SD	62.6 ± 11.5	62.9 ± 13.7		
Median (IQR)	62.9 (55.0–70.7)	63.0 (53.6–71.9)		
Categories, n (%)				
<60	2108 (40.0)	2164 (41.0)		
≥60	3165 (60.0)	3109 (59.0)		
Number of healthcare visits^b^			0.042	−0.028
Mean ± SD	2.8 ± 3.6	2.9 ± 3.6		
Median (IQR)	2.0 (0.0–4.0)	2.0 (1.0–4.0)		
Comorbidities, n (%)^c^				
Gastrointestinal diseases	4525 (85.8)	4550 (86.3)	−0.050	−0.014
Intestinal infections	215 (4.1)	241 (4.6)	0.027	−0.026
Cardiovascular diseases	2719 (51.6)	2884 (54.7)	0.748	−0.070
Obesity/dyslipidemia	399 (7.6)	310 (5.9)	0.340	0.076
Type 1 and type 2 diabetes	676 (12.8)	549 (10.4)	0.434	0.089
Autoimmune diseases	540 (10.2)	502 (9.5)	0.089	0.025
Obstructive sleep apnea	138 (2.6)	128 (2.4)	0.083	0.013
Chronic obstructive pulmonary disease	209 (4.0)	193 (3.7)	0.123	0.018
Myositis	6 (0.1)	3 (0.1)	0.005	0.018
Medications, n (%)^c^				
Antibiotics	3506 (66.5)	3452 (65.5)	0.034	0.022
Proton pump inhibitor	2052 (38.9)	2133 (40.5)	0.159	−0.032
Antiviral drugs	311 (5.9)	320 (6.1)	−0.009	−0.007
IBD medications	4096 (77.7)	3937 (74.7)	−0.119	0.075
Non-aspirin anti-platelet medications	148 (2.8)	156 (3.0)	0.178	−0.011
Aspirin	888 (16.8)	813 (15.4)	0.385	0.045
Non-statin lipid lowering medications	345 (6.5)	408 (7.7)	0.058	−0.050
Anti-diabetic medications	911 (17.3)	762 (14.5)	0.518	0.093
Anticoagulation medications	379 (7.2)	386 (7.3)	0.136	−0.006
Anti-arrhythmic medications	31 (0.6)	27 (0.5)	0.021	0.011

CD: Crohn’s disease; IBD: inflammatory bowel disease; IQR: interquartile range; SD: standard deviation; UC: ulcerative colitis.

a Index date: For statin users, it was defined as date that an individual first attained a cDDD ≥30 for a statin; for non-statin users, it was defined as the same index date as their matched statin users.

b Measured within 2 years and 1 year before the index date.

c Measured within 5 years before the index date. For relevant ICD codes for diseases and ATC codes for medications, see Supplementary Table S3 in the supplementary.

### Incident CRC

**Fig. 3:**
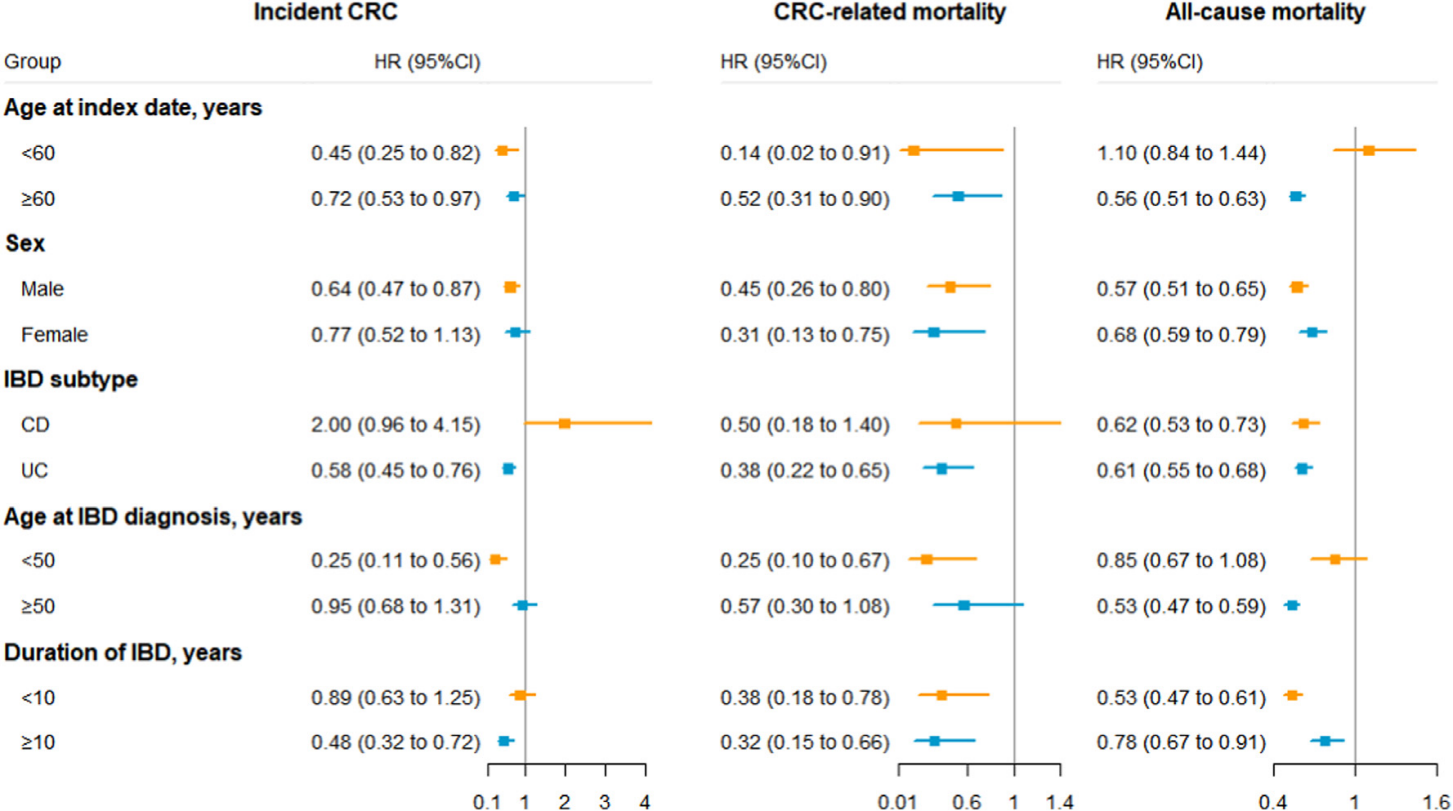
Subgroup analyses of statin use and risk of incident colorectal cancer (CRC), CRC-related mortality, and all-cause mortality in patients with inflammatory bowel disease (IBD). Hazard ratio (HR) and 95% confidence interval (CI) are presented using squares and accompanying horizontal bar. Index date: for statin users, it was defined as date that an individual first attained a cDDD≥30 for a statin; for non-statin users, it was defined as the same index date as their matched statin users. CD: Crohn’s disease; UC: ulcerative colitis.

Statin users had a lower risk of incident CRC than non-statin users (incidence rate (IR): 21.2 vs. 29.2 per 10,000 person-years with a rate difference (RD) of −8.0 (95% CIs: −15.8 to −0.2); aHR = 0.76, 95% CIs: 0.61 to 0.96) (Table 2). In stratified analyses (Fig. 3 and Supplementary Table S5), the inverse association appeared stronger among patients who had initiated statin treatment at younger age (aHR at <60 years = 0.45 (0.25 to 0.82); aHR at ≥60 years = 0.72 (0.53 to 0.97), *P*_for interaction_ = 0.0020), among patients diagnosed with IBD at ages younger than 50 (aHR at <50 years = 0.25 (0.11 to 0.56); aHR at ≥50 years = 0.95 (0.68 to 1.31), *P*_for interaction_ < 0.0001), and among patients with longer disease duration (aHR for ≥10 years = 0.48 (0.32 to 0.72); aHR for <10 years = 0.89 (0.63 to 1.25), *P*_for interaction_ = 0.0090). In addition, this inverse association was stronger in patients diagnosed with IBD in earlier calendar periods (aHR in 1969–1999 = 0.49 (0.32 to 0.73); aHR in 2000–2018 = 0.85 (0.63 to 1.15), *P*_for interaction_ = 0.0060) (Supplementary Table S5).

**Table 2:** Statin use and risk of incident CRC, CRC-related mortality, and all-cause mortality in patients with inflammatory bowel disease.

Outcomes	No. Of events (Incidence rate^a^)		Incidence rate difference (95% CI)^a^	HR (95% CI)^b^
	Statin user	Non-statin user		
Incident CRC	70 (21.2)	90 (29.2)	−8.0 (−15.8 to −0.2)	0.76 (0.61 to 0.96)
CRC-related mortality	20 (6.0)	37 (11.9)	−5.9 (−10.5 to −1.2)	0.56 (0.37 to 0.83)
All-cause mortality	520 (156.4)	719 (231.4)	−75.0 (−96.6 to −53.4)	0.63 (0.57 to 0.69)

CI: confidence interval; CRC: colorectal cancer; HR: hazard ratio.

a Calculated as events per 10,000 person-years.

b Adjusted for covariates that included in propensity score matching. See eMethods in the supplementary.

This decreased risk was only observed among patients with UC (aHR = 0.58 (0.45 to 0.76)), rather than CD (aHR = 2.00 (0.96 to 4.15), *P*_for interaction_ = 0.0060), and was driven by colon cancer (aHR = 0.65 (0.49 to 0.85)), rather than rectal cancer (aHR = 1.19 (0.79 to 1.80)) (Supplementary Table S5). Compared with non-statin users, statin users had a lower risk of developing left-sided colon cancer (aHR = 0.57 (0.34 to 0.93)) and early stage CRC (aHR = 0.67 (0.50 to 0.90)) (Supplementary Table S5). No significant interaction was observed in the analysis by sex.

### CRC-related mortality and all-cause mortality

Statin users were at a lower risk of CRC-related mortality than non-statin users (IR: 6.0 vs. 11.9 per 10,000 person-years with a RD of −5.9 (−10.5 to −1.2); aHR = 0.56 (0.37 to 0.83)) (Table 2). This significantly decreased risk was irrespective of age at statin initiation, sex, calendar period at IBD diagnosis, age at IBD diagnosis, and duration of IBD diagnosis, and was only observed in patients with UC (aHR = 0.38 (0.22 to 0.65)), not in patients with CD (aHR = 0.50 (0.18 to 1.40)) (Fig. 3 and Supplementary Table S5).

Statin users also had a lower risk of all-cause mortality than non-statin users (IR: 156.4 vs. 231.4 per 10,000 person-years with a RD of −75.0 (−96.6 to −53.4); aHR = 0.63 (0.57 to 0.69)) (Table 2). This significantly decreased risk was consistently observed across all prespecified subgroups (e.g., aHR = 0.62 (0.53 to 0.73) for CD; aHR = 0.61 (0.55 to 0.68) for UC), except for patients initiating statin treatment at age <60 years or patients diagnosed with IBD at age <50 years (Fig. 3 and Supplementary Table S5).

### Secondary analyses

In as-treated analysis, statin users were also at a lower risk of incident CRC (aHR = 0.78 (0.60 to 1.01)), CRC-related mortality (aHR = 0.07 (0.02 to 0.28)), and all-cause mortality (aHR = 0.53 (0.47 to 0.60)) compared with non-statin users (Supplementary Table S6). However, since only 2 statin users died from CRC, the finding for CRC-related mortality (aHR = 0.07) merits cautious interpretation. The negative associations between statin use and risk of three outcomes were also observed without considering competing events in the analyses (Supplementary Table S6).

The inverse relationship between statin use and risk of CRC appeared to be duration-dependent, even after restricting the analysis to statin users. Compared with short-term use (30 days–<1 year), the aHRs of incident CRC were 0.59 (0.25 to 1.43) for 1 to <2 years of use, 0.46 (0.21 to 0.98) for 2 to <5 years of use, and 0.38 (0.16 to 0.86) for ≥5 years of use (*P*_for tread_ = 0.016). A significant duration-dependent benefit was also observed for all-cause mortality (*P*_for tread_ < 0.0001), but not for CRC-related mortality (*P*_for tread_ = 0.35). However, the point estimates for CRC-related mortality were less than one in the groups with 2 to <5 years and ≥5 years of use (aHR = 0.61 and 0.62, respectively) (Supplementary Table S6).

### Sensitivity analyses

Our results were consistent across all sensitivity analyses, including those performed when we modeled covariates as time-varying variables (aHR for incident CRC = 0.64 (0.45 to 0.91); aHR for CRC-related mortality = 0.45 (0.25 to 0.81); and aHR for all-cause mortality = 0.62 (0.54 to 0.71)); after we excluded patients with CVD before statin initiation (aHR for incident CRC = 0.56 (0.37 to 0.83); aHR for CRC-related mortality = 0.30 (0.11 to 0.85); and aHR for all-cause mortality = 0.73 (0.60 to 0.90)); after we excluded patients with diabetes before statin initiation (aHR for incident CRC = 0.76 (0.58 to 0.98); aHR for CRC-related mortality = 0.37 (0.21 to 0.65); and aHR for all-cause mortality = 0.67 (0.60 to 0.75)); and after we discarded the first one or two years of follow-up from the analysis (Supplementary Table S7). The estimated E-values were 1.76, 2.97, and 2.55 for the point estimates of incident CRC, CRC-related mortality, and all-cause mortality, respectively. Finally, we did not observe any significant association between statin use and incident fracture in the negative control analysis after adjustment for propensity score (aHR = 1.03 (0.95 to 1.12)) (Supplementary Table S7).

## Discussion

In this nationwide cohort, statin use was associated with a lower risk of incident CRC, CRC-related mortality, and all-cause mortality. The NNT to avoid one incident CRC, CRC-related death, and any death within 10 years after statin initiation was 227, 200, and 21, respectively. The benefits were duration-dependent, with a significantly lower risk after ≥2 years of use. The inverse association for incident CRC and CRC-related mortality were only observed in patients with UC, in patients diagnosed with IBD at age <50 years, in patients with longer IBD duration, for CRC-related mortality in colon cancer, and in early-stage CRC. The benefits for all-cause mortality were consistently observed regardless of sex, IBD subtype, age at IBD diagnosis, and disease duration, suggesting the benefits of using statin may apply to a broad at-risk population.

Among the general population, the chemopreventive effect of statin on CRC has been greatly investigated, the findings have however been contradictive.^13^, ^14^, ^15^, ^16^^,^^34^, ^35^, ^36^, ^37^ In a recent umbrella systematic review and meta-analysis published in 2019 that included 59 studies (46 observational studies and 13 randomized clinical trials),^13^ statin use was associated with a reduced risk of CRC in observational studies (pooled relative risk = 0.92 (0.88–0.95)), but the association was not significant in randomized clinical trials (pooled relative risk = 0.92 (0.81–1.05)). Although these results were consistent with findings from three other meta-analyses published in 2010^16^ and 2014,^14^^,^^15^ more discrepant findings have been reported recently.^34^, ^35^, ^36^, ^37^ Although immortal time bias and including prevalent statin users may partially explain the difference between observational versus randomized effect estimates,^35^ short study periods and short statin exposure periods also raise the concern of whether previous clinical trials had enough follow-up time and adequate statistical power to observe any potential benefits on CRC.

Among IBD patients, four studies until now have investigated potential benefits of statin use on CRC, but with inconclusive results (see Supplementary Table S1 for detail).^17^ Three of them found no association between statin use and CRC risk: aHR = 0.48 (0.14 to 1.59) in a Chinese cohort study of 2103 IBD patients^17^; aHR = 0.56 (0.66 to 4.92) in a cohort study of 642 IBD patients undergoing surveillance colonoscopies^18^; and adjusted odds ratio = 0.10 (0.01 to 1.31) in a case-control study including 1,921CRC cases and 1921 matched controls in Israel.^20^ However, very few CRC cases were observed among statin users in those three studies (e.g., only 6 cases in each of two studies^17^^,^^18^), limiting the power to detect any signal. On the contrary, in a cohort study of 11,001 IBD patients from two tertiary referral hospitals (including 1376 statin users), the authors showed a reduced CRC risk among statin users (adjusted odds ratio = 0.42 (0.28–0.62)),^19^ which was consistent with our finding. Several reasons contribute to this heterogeneity, including different measurements of statin use (e.g., from electronic prescriptions^18^ vs. self-reported^20^), mean/median age at statin use (from 59.4^18^ to 67^19^ years), percentage of male individuals (from 51.4%^20^ to 60.3%^17^), inclusion of prevalent CRC cases,^18^ varied inclusion criteria,^18^ and varied adjusted covariates. Moreover, not all patients with CRC were equally vulnerable to statin therapy due to highly heterogeneous responses, as evidenced by varying responses across different cancer cell lines or in patients with different genetic variations.^38^

Potential mechanisms underlying the chemopreventive effect of statins on CRC were discussed in the Supplementary Material.

Our study benefits from an unselected population, in which information regarding IBD, statin treatment, CRC, and relevant covariates were prospectively collected over a virtually complete follow-up. These factors facilitated the execution of informative subgroup and sensitivity analyses, and thus providing more compelling evidence of an inverse and duration-dependent association between statin use and three outcomes. Such subgroup analyses are important since they can guide clinicians in IBD treatment and optimize personalized medicine for preventing CRC. Given the unselected nationwide population from Sweden with universal health-care access, our study's internal validity is high. In addition, we applied numerous approaches to address potential biases: (i) implementing of a new-user design to minimize selection bias, (ii) discarding 1 or 2 years between statin initiation and outcomes to minimize reverse causation, (iii) utilizing time-varying analysis to minimize misclassification of exposures and covariates, and (iv) applying the negative control outcome analysis to assess potential biases in the study. Moreover, high PPVs^24^^,^^39^ in the registers additionally reduced risks of selection or information bias.

Limitations of the present study should also be acknowledged. First, misclassification of statin use may arise due to the absence of information regarding actual adherence or use of statins during hospitalization. Second, we lacked data on other risk factors for CRC, including lifestyle factors (e.g., smoking, physical inactivity, alcohol, and high red meat consumption) and genetics,^40^ leaving residual confounding as a concern. However, the results were generally consistent and robust across multiple approaches and the estimated E-values suggested that an unmeasured confounder would have to be both strongly associated with CRC and highly imbalanced between statin users and non-statin users to fully attenuate the relationship between statin use and CRC risk. Third, despite being nationwide, our study still suffered from limited statistical power, which hindered us from exploring the potential effect of individual drugs and their associations with tumour stages. Moreover, relatively small numbers of patients with CD resulted in some subgroups being underpowered to reveal real associations with small magnitude. Pooling study populations across countries may circumvent this limitation in the future. Fourth, due to data unavailability, IBD severity was not considered when performing the propensity score matching process and the exploration of the role of statin use in relation to IBD medications (e.g., biologics) was not feasible. Fifth, because we cannot exclude the possibility of a healthy user bias associated with statin use and indication bias that may have been introduced because indications for statin use were not assessable, our results are merely suggestive and cannot prove causality. Finally, given the fact that Swedish healthcare system provides free universal access to healthcare services, generalizability of our findings to other settings should be further cautious due to variations in the use of statin and epidemiological profile of IBD and CRC among countries and ethnicities.

Although statin therapy (long-term and at high dose) has been linked to a slightly increased risk of new-onset diabetes (around 9%)^41^ and muscle symptoms (e.g., pain and weakness, around 3%),^42^ the excess risk of both adverse events is greatly outweighed by the known cardiovascular benefits.^43^^,^^44^ Due to their efficacy in the primary and secondary prevention of cardiovascular morbidity and mortality, statins are one of the most commonly prescribed drugs worldwide.^45^ As summarized in a previous review,^46^ several clinical trials have reported potential benefits of using statins as adjuvants for cancer therapy among patients with cancer. Although further research is needed to define the optimal timing of initiation, exact dose, and minimum duration required to achieve benefits before safely incorporating statin into guidelines for CRC prevention in patients with IBD, our findings indeed provide evidence for the clinical use of statin as a well-tolerated and affordable cancer chemopreventive agent in patients with IBD.

In conclusion, our findings indicated that in IBD patients, statin use was associated with a lower risk of incident CRC, CRC-related mortality, and all-cause mortality. The benefits were duration-dependent and observed primarily in individuals with UC.

## Contributors

Study concept and design: JFL and JS. Acquisition of data: JFL. Drafting of the manuscript: JS and JFL. Interpretation of data, and critical revision of the manuscript for important intellectual content: JS, JH, DB, FE, BR, PL, MS, OO, and JFL. Statistical analysis: JS. Funding acquisition: JFL. Administrative, technical, or material support: JFL. Guarantors: JS and JFL have directly accessed and verified the underlying data reported in the manuscript and take responsibility for the integrity of the data and the accuracy of the data analysis. All authors read and approved the final version of the manuscript.

## Data sharing statement

The data set cannot be shared directly under current legislation for data protection and must be requested directly from the respective registry holders, Statistics Sweden (information@scb.se) and the Swedish National Board of Health and Welfare (registerservice@socialstyrelsen.se), after approval by the Swedish Ethical Review Authority.

## Declaration of interests

All authors have completed the ICMJE uniform disclosure form and declare: JH served as speaker and/or advisory board member for AbbVie, Aqilion, BMS, Celgene, Celltrion, Dr Falk Pharma and the Falk Foundation, Ferring, Galapagos, Gilead, Hospira, Index Pharma, Janssen, MEDA, Medivir, MSD, Novartis, Pfizer, Prometheus Laboratories Inc., Sandoz, Shire, Takeda, Thermo Fisher Scientific, Tillotts Pharma, Vifor Pharma and received grant support from Janssen, MSD and Takeda. PL is an employee of GSK (received and purchased GSK stock). OO has been PI on projects at Karolinska Institutet financed by grants from Janssen, Pfizer, AbbVie, Takeda, Bristol Myer Squibb and Ferring, and Karolinska Institutet has received fees for lectures and participation on advisory boards from Janssen, Ferring, Galapagos, Bristol Myer Squibb, Takeda, and Pfizer. OO also reports grants from Pfizer, Janssen, Galapagos, and AbbVie in the context of a national safety monitoring programs. JFL has coordinated a study on behalf of the Swedish IBD quality register (SWIBREG). That study received funding from Janssen corporation. JFL has also received financial support from MSD developing a paper reviewing national healthcare registers in China. The other authors report no conflict of interest.

## Appendix ASupplementary data

Supplementary Tables S1–S7

R1 Statin CRC protocol
