# Supplementary material for: Statin use and risk of colorectal cancer in patients with inflammatory bowel disease
Source: eClinicalMedicine. 2023 Aug 24;63:102182. doi: 10.1016/j.eclinm.2023.102182 (PMC10474364; doi:10.1016/j.eclinm.2023.102182)
Supplement: Supplementary Tables S1–S7 [file mmc1.docx]

**Statin use and risk of colorectal cancer in patients with inflammatory bowel disease**

Sun J et al.

Contents

[**eMethods** 2](#_Toc139619794)

[**Mechanisms** 2](#_Toc139619795)

[Table S1. Previous studies of statin use and colorectal cancer (CRC) in patients with IBD compared to current study 3](#_Toc139619796)

[Table S2. International Classification of Disease (ICD) codes and SNOMED codes for defining IBD 4](#_Toc139619797)

[Table S3. Definitions of statin, outcomes, exclusion criteria, comorbidities, and medications 5](#_Toc139619798)

[Table S4. Characteristics of statin users and non-statin users before propensity score matching 7](#_Toc139619799)

[Table S5. Subgroup analyses of statin use and risk of incident CRC, CRC-related mortality, and all-cause mortality in patients with inflammatory bowel disease 8](#_Toc139619800)

[Table S6. Secondary analyses of statin use and risk of incident CRC, CRC-related mortality, and all-cause mortality in patients with inflammatory bowel disease 10](#_Toc139619801)

[Table S7. Sensitivity analyses of statin use and risk of incident CRC, CRC-related mortality, and all-cause mortality in patients with inflammatory bowel disease 11](#_Toc139619802)

[References 12](#_Toc139619803)

# **eMethods**

1. **Data source**

ESPRESSO^1^ contains data on GI-related biopsies from all 28 pathology departments in Sweden from 1965 to 2017. The Total Population Register^2^ contains details on birth, death, and migration, which was initiated in 1968. The Prescribed Drug Register^3^ collects information on medications dispensed at all pharmacies in Sweden since July 2005, including dispensing date, drug name, and a defined daily dose (DDD, a standardized measure of average daily drug consumption by the World Health Organization). The National Patient Register^4^ covers data on inpatient (since 1964) and outpatient (since 2001) specialist care with disease diagnoses and surgical procedures. The Cancer Register^5^ contains data from >96% of incident cancer cases since 1958. The Cause of Death Register^6^ contains information on >99% of death since 1952. The Swedish Longitudinal Integrated Database for Health Insurance and Labour Market Studies^7^ contains information on demographic and socioeconomic characteristics (e.g., education and county of residence).

1. **Matching**

Matching was performed in two steps: 1). direct matching and 2). 1:1 propensity score matching. The following covariates were considered.

1. Direct match

- Age at IBD diagnosis, years (<18, 18-<40, 40-<60, and ≥60);
- Sex (male or female);
- IBD subtype (CD or UC);
- Calendar year at IBD diagnosis (1965-1989, 1990-1999, 2000-2009, 2010-2018)

1. 1:1 propensity score matching (nearest-neighbor algorithm without replacement)

- Age at IBD diagnosis, years (continuous);
- Duration of IBD, years (continuous);
- Number of healthcare visits (continuous);
- Country of birth (Nordic countries or others);
- Education (0-9, 10-12, ≥13 years, or missing);
- Comorbidities (yes/no, see Table S3 for detail): gastrointestinal diseases, intestinal infections, cardiovascular diseases, obesity/dyslipidemia, type 1 and type 2 diabetes, autoimmune diseases, obstructive sleep apnea, chronic obstructive pulmonary disease, and myositis;
- Medications (yes/no, see Table S3 for detail): antibiotics, proton pump inhibitor, antiviral medications, IBD medications, non-aspirin anti-platelet medications, aspirin, non-statin lipid lowering medications, anti-diabetic medications, anticoagulation medications, and anti-arrhythmic medications.

# **Mechanisms**

Although the precise mechanisms underlying the chemopreventive effect of statins on CRC are still not fully understood, several explanations have been proposed^8,9^. Statins exert anti-cancer effects by inhibiting the rate-limiting enzyme of the mevalonate pathway and then reducing mevalonate synthesis and lowering cholesterol levels^9^. Increased mevalonate pathway activity has been observed in carcinogenic lesions and suggested as a hallmark of tumorigenesis. The depletion of cholesterol induces apoptosis and inhibits cancer cell growth^8^. In line with that, a recent UK Biobank study suggested that high serum levels of lipids were associated with an increased CRC risk in nonusers of cholesterol-lowering medications^10^. Moreover, statins could reduce the synthesis of nonsterol isoprenoids which play a role in rescuing statin-induced apoptosis^11^. In addition, statins have a number of other pleiotropic effects that may influence tumorigenesis through anti-proliferation, regulating autophagy/apoptosis, and induction of ferroptosis and pyroptosis^9^. For example, as suggested in previous cell line studies, both simvastatin and atorvastatin play a role in inducing CRC cell apoptosis^12,13^.

| Table S1. Previous studies of statin use and colorectal cancer (CRC) in patients with IBD compared to current study | | | | | |
| --- | --- | --- | --- | --- | --- |
| First author, publication year, place, study period | Sample size | Male (%), mean age (years), and follow-up time (years) | No. and incidence rate of CRC | Exposure measurement | Main findings |
| Mak, 2020, China (Hong Kong), a territory-wide registry, Jan 2000-Sep 2016 ^14^ | 2103 IBD patients (857 CD, 1246 UC) | 60.3%, 40.0 yrs, 7.6 yrs | n=6, NA | Not clear | Statin was not associated with a reduced risk of cancer development, hazard ratio=0.48 (0.14-1.59) |
| Shah, 2019, USA, a tertiary referral center, 2005-2016 ^15^ | 642 IBD patients (57 statin users vs. 585 non-statin users) | 54.4% vs. 50.6%, 59.4 yrs vs. 39.5 yrs, 5.0 yrs vs. 3.5 yrs | n=6, NA | Any statin use >3 months | No significant association between statin use and CRC, hazard ratio=0.56 (0.06-4.92) |
| Ananthakrishnan, 2016, USA, a cohort based at two referral hospitals, 1998-2010 ^16^ | 11001 IBD patients (1376 statin user vs. 9625 non-statin user) | 55% vs. 46%, 67 yrs vs. 42 yrs, NA | Statin users: n=30; non-statin users: n=287; NA | Having a statin prescription for > 6 months before diagnosis of CRC or the end of follow-up | Statin was inversely associated with CRC: odds ratio=0.42 (0.28-0.62) |
| Samadder, 2011, Israel, Molecular Epidemiology of Colorectal Cancer study, March 1998-July 2004 ^17^ | 1921 CRC cases and 1921 individually matched controls | 51.4%, 69.8 yrs vs. 70.4 yrs, NA | NA | Self-reported | Statin use for at least 5 years was not associated with an reduced risk of IBD-associated CRC, odds ratio=0.10 (0.01-1.31) |
| Current study | 5273 new users of statin and 5,273 matched non-statin users | 56.9%, 51.7 yrs, 5.6 yrs | 160 incident CRC; users: n=70 (incident rate=21.2 per 10000 person-years); non-statin users: n=90 (incident rate=29.2) | Identified from the Prescribed Drug Register; statin users were identified as those aged ≥ 18 years and with a statin prescription for ≥30 cumulative DDDs (cDDDs) from July, 2006 or later; the date that an individual first attained a cDDD ≥ 30 for a statin was defined as the index date for statin exposure | Statin users had a lower risk of incident CRC than non-statin users (hazard ratio=0.76, 95%CI: 0.61 to 0.96); the benefit was duration-dependent, with a significantly lower risk after ≥2 years of use; the benefit was observed in patients with UC, but not in patients with CD |
| NA: not available | | | | | |

| Table S2. International Classification of Disease (ICD) codes and SNOMED codes for defining IBD | | | | | |
| --- | --- | --- | --- | --- | --- |
| IBD subtypes ^a^ | ICD-7 (1964-1968) | ICD-8 (1969-1986) | ICD-9 (1987-1996) | ICD-10 (1997-) | SNOMED codes ^b^ |
| Ulcerative colitis (UC) | 572,20; 572,21; 578,03 | 563,1; 563,10; 569,02; 569,04 | 556 | K51 | D6255 or M41, M42, M43, M44, M463, or M47 |
| Crohn's disease (CD) | 572,00; 572,09 | 563,00 | 555 | K50 | D6216 or M41, M42, M43, M44, M463, or M47 |
| IBD unclassified (IBD-U) | UC + CD | UC + CD or 563; 563,0; 563,9; 563,98; 563,99 | UC + CD | UC + CD or K52.3 | D6214 or M41, M42, M43, M44, M463, or M47 |
| ^a^ Subtypes of IBD were defined according to the first two diagnostic codes only, therefore no information after start of follow-up contributed to such definition; and the IBD subtype was only determined by the ICD code if one individual had one ICD code for IBD and one M code. ≥1 ICD code for IBD plus a relevant biopsy code has a positive predictive value of 95% ^18,19^. In a recent paper ^20^, we report that 18% of incident IBD patients in the Swedish patient register during 2002-2014 were classified as another IBD subtype at some point during follow-up. | | | | | |
| ^b^ SNOMED codes starting with M (inflammation suggestive of IBD, but not a specified subtype) were required to be accompanied by a topographic code of T67 or T68 (colon); for example, M41 refers to all codes starting with M41; D codes are diagnostic codes but listed under morphology in pathology registers; D6255, for example, is the diagnostic code for UC. | | | | | |

| Table S3. Definitions of statin, outcomes, exclusion criteria, comorbidities, and medications | |
| --- | --- |
| **Statin** | **ATC code** ^a^ |
| Simvastatin | C10AA01 |
| Pravastatin | C10AA03 |
| Atorvastatin | C10AA05 |
| Rosuvastatin | C10AA07 |
| **Outcomes** | **ICD code** ^a^ |
| Incident colorectal cancer (CRC) ^b^ | ICD-7: 153-154, but not 1541 (anal cancer) or 1534 (appendiceal cancer) |
| CRC-related mortality | ICD-10: C18-20, but not C181 (appendiceal cancer) |
| All-cause mortality | ICD-10: A00-U99 |
| **Proctocolectomy and colectomy** | **Procedure code** |
| Proctocolectomy ^c^ |  |
|  | Sixth version (1969-1996): 4652, 4653, 4654 |
|  | Seventh version (1997-): JFH20-JFH40 |
| Colectomy with an intact rectum ^d^ |  |
|  | Sixth version (1969-1996): 4650, 4651 |
|  | Seventh version (1997-): JFH00, JFH01, JFH10, JFH11, JFH96 |
| Rectum amputation after a colectomy with an intact rectum ^e^ |  |
|  | Sixth version (1969-1996): 4820, 4821, 4822, 4828 |
|  | Seventh version (1997-): JGB00-JGB97 |
| **Exclusion criteria** | **ICD-10 code/procedure code** |
| Cancer | C00-C97, except non-melanoma skin cancer (C44) |
| HIV/AIDS | B20-B24, F024, O987, R75, Z114, Z219, Z711 |
| Primary immunodeficiency disease | D71, D80–D84 |
| Severe liver failure | B150, B160, B162, B190, K704, K72, K766, I85 |
| End-stage renal disease | N18.5,  N18.6, Z49, Z99.2, Z94.0; Procedure codes: 9200; V9200; 9212; V9212; 9314; V9531; DR012; DR013; DR016; DR024; QF006; 9211; V9211; 9213; V9213; V9532; DR015; DR023; DR055; DV056; 9219; V9219; 9223; V9223; DR017; DR020; DR055; DR056; 6070; KAS10; KAS20 |
| Any organ transplantation | Swedish surgery codes (KVÅ codes): KAS, FQA, FQB, GDG, JJC |
| **Comorbidities** | **ICD-10 code** |
| Gastrointestinal diseases | K00-K99 |
| Intestinal infections | A00-A09 |
| Cardiovascular diseases | I00-I99 |
| Obesity/dyslipidemia | E78 , E65, E66 |
| Type 1 and type 2 diabetes | E10-E14 , O24 |
| Autoimmune diseases | E035, E039, E050, E055, E059, E063, E065, G35, M05-M09, M32, L40 |
| Obstructive sleep apnea | G473 |
| Chronic obstructive pulmonary disease (only if diagnosed ≥40 years) | J41-J44 |
| Myositis (no adequate codes present for rhabdomyolysis) | M60 |
| **Medications** | **ATC code** |
| Antibiotics | J01 |
| Proton pump inhibitor | A02BC |
| Antiviral drugs | J05 |
| IBD medications |  |
| Immunomodulators |  |
| Azathioprine | L04AX01 |
| Mercaptopurine | L01BB02 |
| Methotrexate | L04AX03/L01BA01 |
| Anti-TNF treatment |  |
| Infliximab | L04AB02 (L04AA12 before 2008) |
| Adalimumab | L04AB04 (L04AA17 before 2008) |
| Golimumab | L04AB06 |
| Vedolizumab | L04AA33 |
| Systemic corticosteroids |  |
| Betamethasone | H02AB01 |
| Dexamethasone | H02AB02 |
| Methylprednisolone | H02AB04 |
| Prednisolone | H02AB06 |
| Prednisone | H02AB07 |
| Hydrocortisone | H02AB09 |
| Cortisone | H02AB10 |
| Systemic aminosalicylates (5-ASA) |  |
| Sulfasalazine | A07EC01 |
| Mesalazine | A07EC02 |
| Olsalazine | A07EC03 |
| Balsalazide | A07EC04 |
| Rectal aminosalicylates (5-ASA) |  |
| Mesalazine | A07EC02 |
| Corticosteroids acting locally |  |
| Hydrocortisone | A07EA02 |
| Budesonide | A07EA06 |
| Non-aspirin anti-platelet medications | B01AC excluding aspirin (B01AC06) |
| Aspirin | B01AC06 |
| Non-statin lipid lowering medications | C10AB, C10AC, C10AD, C10AX01-14 |
| Anti-diabetic medications | A10 |
| Anticoagulation medications | B01AA, B01AE, B01AF, B01AX |
| Anti-arrhythmic medications | C01BA, C01BB, C01BC, C01BD, C01BG |
| ATC: Anatomical Therapeutic Chemical; CRC: colorectal cancer; HIV/AIDS: human immunodeficiency virus/ acquired immunodeficiency syndrome; ICD: the International Classification of Disease. | |
| ^a^ ATC codes were identified from the Prescribed Drug Register; ICD codes and procedure codes were identified from the National Patient Register. | |
| ^b^ Incident CRC was ascertained from the Cancer Register, while CRC-related mortality was ascertained from the Cause of Death Register. In the Cancer Register, all ICD-8, ICD-9, and ICD-10 codes were back-translated to ICD-7. Right-sided colon cancer was defined as colon cancer in the ascending colon and hepatic flexure (ICD-10: C182 and C183); left-sided colon cancer was defined as colon cancer in the splenic flexure, descending, and sigmoid colon (ICD-10: C185, C186 and C187). While the two proximal thirds of the transverse colon is usually included in right-sided colon, in the analysis by the location of colon cancer, we did not include colon cancer cases from the transverse colon, since our data did not allow distinction between right-sided or left-sided transverse colon cancer. | |
| ^c^ Not at risk of colorectal cancer. | |
| ^d^ Only at risk of rectal cancer. | |
| ^e^ Not at risk of colorectal cancer. | |

| Table S4. Characteristics of statin users and non-statin users before propensity score matching | | |
| --- | --- | --- |
| Characteristic | Statin users (n=5433) | Non-statin users (n=44706) |
| Age at IBD diagnosis, years |  |  |
| Mean ± SD | 51.6 ± 13.3 | 39.6 ± 16.8 |
| Median (IQR) | 51.9 (42.5-60.7) | 36.8 (26.4-50.6) |
| Categories, n (%) |  |  |
| <18 | 23 (0.4) | 2099 (4.7) |
| 18-<40 | 1051 (19.3) | 23274 (52.1) |
| 40-<60 | 2904 (53.5) | 13252 (29.6) |
| ≥60 | 1455 (26.8) | 6081 (13.6) |
| Sex, n (%) |  |  |
| Male | 3109 (57.2) | 21774 (48.7) |
| Female | 2324 (42.8) | 22932 (51.3) |
| Calendar period at IBD diagnosis, n (%) |  |  |
| 1969-1989 | 384 (7.1) | 2039 (4.6) |
| 1990-1999 | 1444 (26.6) | 8560 (19.2) |
| 2000-2009 | 2813 (51.8) | 20288 (45.4) |
| 2010-2018 | 792 (14.6) | 13819 (30.9) |
| IBD subtype, n (%) |  |  |
| CD | 1642 (30.2) | 16212 (36.3) |
| UC | 3791 (69.8) | 28494 (63.7) |
| Country of birth, n (%) |  |  |
| Nordic country | 5016 (92.3) | 40909 (91.5) |
| Others | 417 (7.7) | 3794 (8.5) |
| Educational attainment, n (%) |  |  |
| 0-9 y | 1461 (26.9) | 8310 (18.6) |
| 10-12 y | 2374 (43.7) | 19722 (44.1) |
| ≥13 y | 1168 (21.5) | 12669 (28.3) |
| Missing | 430 (7.9) | 4005 (9.0) |
| Number of healthcare visits ^a^ |  |  |
| Mean ± SD | 2.8 ± 3.6 | 2.8 ± 3.9 |
| Median (IQR) | 2.0 (0.0-4.0) | 2.0 (0.0-4.0) |
| Comorbidities, n (%) ^b^ |  |  |
| Gastrointestinal diseases | 4664 (85.9) | 39444 (88.2) |
| Intestinal infections | 218 (4.0) | 2025 (4.5) |
| Cardiovascular diseases | 2868 (52.8) | 6729 (15.1) |
| Obesity/dyslipidemia | 491 (9.0) | 701 (1.6) |
| Diabetes | 775 (14.3) | 981 (2.2) |
| Autoimmune diseases | 559 (10.3) | 3092 (6.9) |
| Obstructive sleep apnea | 152 (2.8) | 437 (1.0) |
| Chronic obstructive pulmonary disease | 213 (3.9) | 753 (1.7) |
| Myositis | 6 (0.1) | 44 (0.1) |
| Medications, n (%) ^b^ |  |  |
| Antibiotics | 3609 (66.4) | 28447 (63.6) |
| Proton pump inhibitor | 2127 (39.2) | 13336 (29.8) |
| Antiviral drugs | 321 (5.9) | 2665 (6.0) |
| IBD medications | 4204 (77.4) | 36080 (80.7) |
| Non-aspirin anti-platelet medications | 170 (3.1) | 255 (0.6) |
| Aspirin | 949 (17.5) | 2045 (4.6) |
| Non-statin lipid lowering medications | 364 (6.7) | 2579 (5.8) |
| Anti-diabetic medications | 1025 (18.9) | 1129 (2.5) |
| Anticoagulation medications | 390 (7.2) | 1353 (3.0) |
| Anti-arrhythmic medications | 31 (0.6) | 111 (0.3) |
| CD: Crohn’s disease; IBD: inflammatory bowel disease; IQR: interquartile range; SD: standard deviation; UC: ulcerative colitis. | | |
| ^a^ Measured within 2 years and 1 year before the index date. Index date: for statin users, it was defined as date that an individual first attained a cDDD ≥ 30 for a statin; for non-statin users, it was defined as the same index date as their matched statin users. | | |
| ^b^ Measured within 5 years before the index date. | | |

| Table S5. Subgroup analyses of statin use and risk of incident CRC, CRC-related mortality, and all-cause mortality in patients with inflammatory bowel disease | | | | | |
| --- | --- | --- | --- | --- | --- |
|  | No. of events (Incidence rate ^a^) | | Incidence rate difference (95%CI) ^a^ | HR (95%CI) ^b^ | *P* for interaction |
|  | Statin user | Non-statin user |  |  |  |
| **Incident CRC** | | | | | |
| Age at index date, years |  |  |  |  | 0.0020 |
| <60 | 11 (7.6) | 27 (18.7) | -11.1 (-19.4 to -2.7) | 0.45 (0.25 to 0.82) |  |
| ≥60 | 59 (31.8) | 63 (38.5) | -6.7 (-19.2 to 5.8) | 0.72 (0.53 to 0.97) |  |
| Sex |  |  |  |  | 0.25 |
| Male | 41 (21.4) | 59 (33.5) | -12.1 (-22.9 to -1.3) | 0.64 (0.47 to 0.87) |  |
| Female | 29 (20.8) | 31 (23.4) | -2.6 (-13.8 to 8.6) | 0.77 (0.52 to 1.13) |  |
| Calendar period at IBD diagnosis | |  |  |  | 0.0060 |
| 1969-1999 | 23 (19.7) | 43 (39.3) | -19.6 (-33.8 to -5.3) | 0.49 (0.32 to 0.73) |  |
| 2000-2018 | 47 (22.0) | 47 (23.7) | -1.7 (-10.9 to 7.6) | 0.85 (0.63 to 1.15) |  |
| Educational attainment |  |  |  |  | 0.28 |
| 0-9 y | 26 (29.4) | 33 (38.5) | -9.1 (-26.4 to 8.3) | 0.31 (0.14 to 0.70) |  |
| 10-12 y | 25 (17.1) | 35 (26.5) | -9.3 (-20.4 to 1.7) | 0.72 (0.43 to 1.20) |  |
| ≥13 y | 14 (19.9) | 10 (16.0) | 3.8 (-10.5 to 18.2) | 1.33 (0.59 to 3.01) |  |
| Missing | 5 (19.4) | 12 (43.3) | -24.0 (-53.8 to 5.9) | 0.57 (0.23 to 1.41) |  |
| IBD subtype |  |  |  |  | 0.0060 |
| CD | 13 (13.4) | 7 (7.7) | 5.8 (-3.5 to 15.0) | 2.00 (0.96 to 4.15) |  |
| UC | 57 (24.4) | 83 (38.3) | -13.9 (-24.3 to -3.5) | 0.58 (0.45 to 0.76) |  |
| Age at IBD diagnosis, years |  |  |  |  | <0.0001 |
| <50 | 13 (8.8) | 42 (28.2) | -19.4 (-29.2 to -9.6) | 0.25 (0.11 to 0.56) |  |
| ≥50 | 57 (31.1) | 48 (30.1) | 1.0 (-10.8 to 12.7) | 0.95 (0.68 to 1.31) |  |
| Duration of IBD, years |  |  |  |  | 0.0090 |
| <10 | 44 (23.3) | 42 (24.5) | -1.2 (-11.3 to 8.9) | 0.89 (0.63 to 1.25) |  |
| ≥10 | 26 (18.4) | 48 (35.1) | -16.7 (-28.9 to -4.5) | 0.48 (0.32 to 0.72) |  |
| Lipophilic statin |  |  |  |  | - |
| Simvastatin | 56 (22.2) | 71 (30.5) | -8.3 (-17.5 to 0.9) | 0.68 (0.52 to 0.90) |  |
| Atorvastatin | 14 (19.0) | 19 (26.7) | -7.7 (-23.2 to 7.9) | 0.68 (0.41 to 1.14) |  |
| CRC subtype |  |  |  |  | - |
| Colon cancer | 45 (13.6) | 69 (22.4) | -8.8 (-15.4 to -2.2) | 0.65 (0.49 to 0.85) |  |
| Rectal cancer | 25 (7.6) | 21 (6.8) | 0.7 (-3.4 to 4.9) | 1.19 (0.79 to 1.80) |  |
| Colon cancer |  |  |  |  | - |
| Left-sided | 13 (3.9) | 23 (7.5) | -3.5 (-7.3 to 0.2) | 0.57 (0.34 to 0.93) |  |
| Right-sided | 15 (4.5) | 19 (6.2) | -1.6 (-5.2 to 2.0) | 0.79 (0.49 to 1.28) |  |
| Cancer stage (TNM) ^c^ |  |  |  |  | - |
| I-II | 39 (11.8) | 57 (18.5) | -6.7 (-12.8 to -0.6) | 0.67 (0.50 to 0.90) |  |
| III-IV | 24 (7.3) | 25 (8.1) | -0.9 (-5.2 to 3.5) | 0.96 (0.65 to 1.43) |  |
| **CRC-related mortality** | | | | | |
| Age at index date, years |  |  |  |  | 0.0020 |
| <60 | 2 (1.4) | 9 (6.2) | -4.8 (-9.3 to -0.3) | 0.14 (0.02 to 0.91) |  |
| ≥60 | 18 (9.6) | 28 (17.0) | -7.4 (-15.0 to 0.3) | 0.52 (0.31 to 0.90) |  |
| Sex |  |  |  |  | 0.17 |
| Male | 15 (7.8) | 22 (12.4) | -4.6 (-11.1 to 1.9) | 0.45 (0.26 to 0.80) |  |
| Female | 5 (3.6) | 15 (11.3) | -7.7 (-14.2 to -1.2) | 0.31 (0.13 to 0.75) |  |
| Calendar period at IBD diagnosis | |  |  |  | 0.83 |
| 1969-1999 | 9 (7.7) | 17 (15.3) | -7.7 (-16.5 to 1.2) | 0.50 (0.27 to 0.94) |  |
| 2000-2018 | 11 (5.1) | 20 (10.0) | -4.9 (-10.2 to 0.4) | 0.32 (0.15 to 0.66) |  |
| Educational attainment |  |  |  |  | - |
| 0-9 y | 10 (11.2) | 14 (16.2) | -4.9 (-15.9 to 6.0) | 0.38 (0.13 to 1.05) |  |
| 10-12 y | 5 (3.4) | 9 (6.7) | -3.3 (-8.7 to 2.0) | 0.80 (0.31 to 2.04) |  |
| ≥13 y | 4 (5.7) | 3 (4.8) | 0.9 (-6.9 to 8.6) | - |  |
| Missing | 1 (3.9) | 11 (39.3) | -35.4 (-59.8 to -11.0) | - |  |
| IBD subtype |  |  |  |  | 0.67 |
| CD | 4 (4.1) | 6 (6.5) | -2.4 (-9.0 to 4.2) | 0.50 (0.18 to 1.40) |  |
| UC | 16 (6.8) | 31 (14.2) | -7.4 (-13.4 to -1.4) | 0.38 (0.22 to 0.65) |  |
| Age at IBD diagnosis, years |  |  |  |  | 0.95 |
| <50 | 5 (3.4) | 19 (12.6) | -9.3 (-15.7 to -2.8) | 0.25 (0.10 to 0.67) |  |
| ≥50 | 15 (8.1) | 18 (11.2) | -3.1 (-9.7 to 3.5) | 0.57 (0.30 to 1.08) |  |
| Duration of IBD, years |  |  |  |  | 0.25 |
| <10 | 12 (6.3) | 17 (9.8) | -3.5 (-9.4 to 2.3) | 0.38 (0.18 to 0.78) |  |
| ≥10 | 8 (5.6) | 20 (14.5) | -8.9 (-16.3 to -1.4) | 0.32 (0.15 to 0.66) |  |
| Lipophilic statin |  |  |  |  | - |
| Simvastatin | 14 (5.5) | 29 (12.3) | -6.8 (-12.2 to -1.5) | 0.32 (0.18 to 0.58) |  |
| Atorvastatin | 6 (8.1) | 8 (11.2) | -3.0 (-13.1 to 7.1) | 0.71 (0.31 to 1.63) |  |
| **All-cause mortality** | | | | | |
| Age at index date, years |  |  |  |  | 0.0010 |
| <60 | 75 (51.6) | 76 (52.2) | -0.5 (-17.1 to 16.0) | 1.10 (0.84 to 1.44) |  |
| ≥60 | 445 (237.7) | 643 (389.6) | -152.0 (-189.3 to -114.6) | 0.56 (0.51 to 0.63) |  |
| Sex |  |  |  |  | 0.043 |
| Male | 299 (155.2) | 448 (252.1) | -96.9 (-126.1 to -67.7) | 0.57 (0.51 to 0.65) |  |
| Female | 221 (158.1) | 271 (203.8) | -45.7 (-77.7 to -13.7) | 0.68 (0.59 to 0.79) |  |
| Calendar period at IBD diagnosis | |  |  |  | <0.0001 |
| 1969-1999 | 223 (190.1) | 263 (237.4) | -47.3 (-85.4 to -9.3) | 0.76 (0.65 to 0.87) |  |
| 2000-2018 | 297 (138.1) | 456 (228.1) | -90.0 (-116.2 to -63.9) | 0.54 (0.48 to 0.61) |  |
| Educational attainment |  |  |  |  | 0.24 |
| 0-9 y | 232 (260.9) | 293 (338.4) | -77.5 (-128.8 to -26.3) | 0.68 (0.55 to 0.85) |  |
| 10-12 y | 171 (116.5) | 238 (178.4) | -61.9 (-90.5 to -33.3) | 0.64 (0.49 to 0.82) |  |
| ≥13 y | 66 (93.3) | 94 (150.0) | -56.7 (-94.5 to -18.9) | 0.79 (0.45 to 1.38) |  |
| Missing | 51 (196.6) | 94 (335.5) | -138.9 (-225.6 to -52.2) | 0.60 (0.44 to 0.82) |  |
| IBD subtype |  |  |  |  | 0.95 |
| CD | 156 (160.6) | 221 (241.0) | -80.4 (-120.9 to -39.8) | 0.62 (0.53 to 0.73) |  |
| UC | 364 (154.7) | 498 (227.4) | -72.7 (-98.2 to -47.2) | 0.61 (0.55 to 0.68) |  |
| Age at IBD diagnosis, years |  |  |  |  | 0.11 |
| <50 | 100 (67.7) | 122 (81.2) | -13.5 (-33.1 to 6.1) | 0.85 (0.67 to 1.08) |  |
| ≥50 | 420 (227.4) | 597 (372.1) | -144.8 (-181.7 to -107.8) | 0.53 (0.47 to 0.59) |  |
| Duration of IBD, years |  |  |  |  | <0.0001 |
| <10 | 292 (153.4) | 424 (245.5) | -92.1 (-121.4 to -62.8) | 0.53 (0.47 to 0.61) |  |
| ≥10 | 228 (160.4) | 295 (213.8) | -53.3 (-85.4 to -21.3) | 0.78 (0.67 to 0.91) |  |
| Lipophilic statin |  |  |  |  | - |
| Simvastatin | 438 (172.5) | 591 (251.6) | -79.2 (-105.1 to -53.3) | 0.62 (0.56 to 0.68) |  |
| Atorvastatin | 76 (102.8) | 120 (167.4) | -64.6 (-102.4 to -26.7) | 0.62 (0.49 to 0.78) |  |
| CD: Crohn’s disease; CI: confidence interval; CRC: colorectal cancer; HR: hazard ratio; IBD: inflammatory bowel disease; UC: ulcerative colitis. | | | | | |
| ^a^ Calculated as events per 10,000 person-years. | | | | | |
| ^b^ Adjusted for covariates that included in propensity score matching. See eMethods in the supplementary. | | | | | |
| ^c^ Cancer stage by type of tumor, presence of cancer in lymph nodes, or presence of metastases (TNM). The TNM staging was based on the clinical TNM classification. | | | | | |

| Table S6. Secondary analyses of statin use and risk of incident CRC, CRC-related mortality, and all-cause mortality in patients with inflammatory bowel disease | | | | |
| --- | --- | --- | --- | --- |
| Secondary analyses | No. of events (Incidence rate ^a^) | | Incidence rate difference (95%CI) ^a^ | HR (95%CI) ^b^ |
|  | Statin user | Non-statin user |  |  |
| **As-treated analysis** | | | | |
| Incident CRC | 50 (23.8) | 90 (29.2) | -5.5 (-14.4 to 3.5) | 0.78 (0.60 to 1.01) |
| CRC-related mortality | 2 (1.0) | 37 (11.9) | -11.0 (-15.0 to -6.9) | 0.07 (0.02 to 0.28) |
| All-cause mortality | 295 (140.2) | 719 (231.4) | -91.3 (-114.5 to -68.0) | 0.53 (0.47 to 0.60) |
| **Without considering competing risk** | | | | |
| Incident CRC | 70 (21.2) | 90 (29.2) | -8.0 (-15.8 to -0.2) | 0.68 (0.49 to 0.95) |
| CRC-related mortality | 20 (6.0) | 37 (11.9) | -5.9 (-10.5 to -1.2) | 0.40 (0.22 to 0.74) |
| All-cause mortality | 520 (156.4) | 719 (231.4) | -75.0 (-96.6 to -53.4) | 0.61 (0.54 to 0.70) |
| **Cumulative duration of statin use** ^c^ | | | | |
|  | **Cases/controls** | **OR (95%CI)** | ***P* _for trend_** |  |
| Incident CRC |  |  |  |  |
| 30 days to <1 year | 20/64 | Ref. |  |  |
| 1 to <2 years | 11/55 | 0.59 (0.25 to 1.43) |  |  |
| 2 to <5 years | 21/122 | 0.46 (0.21 to 0.98) |  |  |
| ≥5 years | 17/89 | 0.38 (0.16 to 0.86) | 0.016 |  |
| CRC-related mortality |  |  |  |  |
| 30 days to <1 year | 3/17 | Ref. |  |  |
| 1 to <2 years | 5/8 | 4.89 (0.33 to 71.52) |  |  |
| 2 to <5 years | 6/39 | 0.61 (0.08 to 4.55) |  |  |
| ≥5 years | 6/33 | 0.62 (0.09 to 4.43) | 0.35 |  |
| All-cause mortality |  |  |  |  |
| 30 days to <1 year | 143/313 | Ref. |  |  |
| 1 to <2 years | 93/301 | 0.67 (0.48 to 0.93) |  |  |
| 2 to <5 years | 155/581 | 0.58 (0.43 to 0.79) |  |  |
| ≥5 years | 69/328 | 0.41 (0.27 to 0.62) | <0.0001 |  |
| ^a^ Calculated as events per 10,000 person-years. | | | | |
| ^b^ Adjusted for covariates included in eMethods. | | | | |
| ^c^ Estimated by conditional logistic regression in the nested case-control study, and adjusted for covariates that included in propensity score matching. | | | | |

| Table S7. Sensitivity analyses of statin use and risk of incident CRC, CRC-related mortality, and all-cause mortality in patients with inflammatory bowel disease | | | | |
| --- | --- | --- | --- | --- |
| Sensitivity analyses | No. of events (Incidence rate ^a^) | | Incidence rate difference (95%CI) ^a^ | HR (95%CI) ^b^ |
|  | Statin user | Non-statin user |  |  |
| **Covariates were adjusted as time-varying variables** | | | | |
| Incident CRC | 70 (21.2) | 90 (29.2) | -8.0 (-15.8 to -0.2) | 0.64 (0.45 to 0.91) |
| CRC-related mortality | 20 (6.0) | 37 (11.9) | -5.9 (-10.5 to -1.2) | 0.45 (0.25 to 0.81) |
| All-cause mortality | 520 (156.4) | 719 (231.4) | -75.0 (-96.6 to -53.4) | 0.62 (0.54 to 0.71) |
| **Exclude individuals with CVD before statin initiation** | | | | |
| Incident CRC | 30 (18.1) | 44 (30.2) | -12.1 (-23.1 to -1.1) | 0.56 (0.37 to 0.83) |
| CRC-related mortality | 9 (5.4) | 14 (9.5) | -4.1 (-10.2 to 2.0) | 0.30 (0.11 to 0.85) |
| All-cause mortality | 129 (77.2) | 197 (133.7) | -56.5 (-79.4 to -33.5) | 0.73 (0.60 to 0.90) |
| **Exclude individuals with diabetes before statin initiation** | | | | |
| Incident CRC | 63 (22.0) | 79 (28.0) | -6.0 (-14.2 to 2.2) | 0.76 (0.58 to 0.98) |
| CRC-related mortality | 18 (6.2) | 32 (11.3) | -5.0 (-9.9 to -0.2) | 0.37 (0.21 to 0.65) |
| All-cause mortality | 426 (147.9) | 539 (189.5) | -41.6 (-62.9 to -20.3) | 0.67 (0.60 to 0.75) |
| **Discard the first one year of follow-up from the analysis** | | | | |
| Incident CRC | 60 (21.6) | 74 (28.9) | -7.3 (-15.8 to 1.2) | 0.78 (0.61 to 1.00) |
| CRC-related mortality | 18 (6.4) | 36 (13.9) | -7.5 (-12.9 to -2.1) | 0.49 (0.31 to 0.75) |
| All-cause mortality | 446 (159.2) | 579 (223.7) | -64.5 (-87.9 to -41.0) | 0.66 (0.60 to 0.73) |
| **Discard the first two years of follow-up from the analysis** | | | | |
| Incident CRC | 47 (20.5) | 62 (29.7) | -9.2 (-18.7 to 0.2) | 0.70 (0.53 to 0.93) |
| CRC-related mortality | 15 (6.5) | 28 (13.3) | -6.8 (-12.7 to -0.9) | 0.43 (0.26 to 0.72) |
| All-cause mortality | 378 (163.6) | 483 (229.0) | -65.4 (-91.6 to -39.1) | 0.65 (0.58 to 0.72) |
| **Negative control outcome analysis** | | | | |
| Fracture | 741 (243.4) | 703 (242.7) | 0.7 (-24.4 to 25.8) | 1.03 (0.95 to 1.12) |
| ^a^ Calculated as events per 10,000 person-years. | | | | |
| ^b^ Adjusted for covariates that included in propensity score matching. See eMethods in the supplementary. | | | | |

# References

1. Ludvigsson JF, Lashkariani M. Cohort profile: ESPRESSO (Epidemiology Strengthened by histoPathology Reports in Sweden). Clin Epidemiol 2019;11:101-14.

2. Ludvigsson JF, Almqvist C, Bonamy AK, et al. Registers of the Swedish total population and their use in medical research. Eur J Epidemiol 2016;31:125-36.

3. Wettermark B, Hammar N, Fored CM, et al. The new Swedish Prescribed Drug Register--opportunities for pharmacoepidemiological research and experience from the first six months. Pharmacoepidemiol Drug Saf 2007;16:726-35.

4. Ludvigsson JF, Andersson E, Ekbom A, et al. External review and validation of the Swedish national inpatient register. BMC Public Health 2011;11:450.

5. Barlow L, Westergren K, Holmberg L, Talback M. The completeness of the Swedish Cancer Register: a sample survey for year 1998. Acta Oncol 2009;48:27-33.

6. Brooke HL, Talback M, Hornblad J, et al. The Swedish cause of death register. Eur J Epidemiol 2017;32:765-73.

7. Ludvigsson JF, Svedberg P, Olen O, Bruze G, Neovius M. The longitudinal integrated database for health insurance and labour market studies (LISA) and its use in medical research. Eur J Epidemiol 2019;34:423-37.

8. Longo J, van Leeuwen JE, Elbaz M, Branchard E, Penn LZ. Statins as Anticancer Agents in the Era of Precision Medicine. Clin Cancer Res 2020;26:5791-800.

9. Jiang W, Hu JW, He XR, Jin WL, He XY. Statins: a repurposed drug to fight cancer. J Exp Clin Cancer Res 2021;40:241.

10. Yuan F, Wen W, Jia G, Long J, Shu X-O, Zheng W. Serum lipid profiles and cholesterol-lowering medication use in relation to subsequent risk of colorectal cancer in the UK Biobank cohort. Cancer Epidemiology, Biomarkers & Prevention 2023;32:524-30.

11. Yu R, Longo J, van Leeuwen JE, et al. Statin-induced cancer cell death can be mechanistically uncoupled from prenylation of RAS family proteins. Cancer research 2018;78:1347-57.

12. Wu X, Song M, Qiu P, et al. Synergistic chemopreventive effects of nobiletin and atorvastatin on colon carcinogenesis. Carcinogenesis 2017;38:455-64.

13. Jang HJ, Hong EM, Park SW, et al. Statin induces apoptosis of human colon cancer cells and downregulation of insulin-like growth factor 1 receptor via proapoptotic ERK activation. Oncol Lett 2016;12:250-6.

14. Mak JWY, So J, Tang W, et al. Cancer risk and chemoprevention in Chinese inflammatory bowel disease patients: a population-based cohort study. Scand J Gastroenterol 2020;55:279-86.

15. Shah SC, Glass J, Giustino G, et al. Statin Exposure Is Not Associated with Reduced Prevalence of Colorectal Neoplasia in Patients with Inflammatory Bowel Disease. Gut Liver 2019;13:54-61.

16. Ananthakrishnan AN, Cagan A, Cai T, et al. Statin Use Is Associated With Reduced Risk of Colorectal Cancer in Patients With Inflammatory Bowel Diseases. Clin Gastroenterol Hepatol 2016;14:973-9.

17. Samadder NJ, Mukherjee B, Huang SC, et al. Risk of colorectal cancer in self-reported inflammatory bowel disease and modification of risk by statin and NSAID use. Cancer 2011;117:1640-8.

18. Nguyen LH, Örtqvist AK, Cao Y, et al. Antibiotic use and the development of inflammatory bowel disease: a national case-control study in Sweden. The Lancet Gastroenterology & Hepatology 2020;5:986-95.

19. Mouratidou N, Malmborg P, Jaras J, et al. Identification of Childhood-Onset Inflammatory Bowel Disease in Swedish Healthcare Registers: A Validation Study. Clin Epidemiol 2022;14:591-600.

20. Everhov AH, Sachs MC, Malmborg P, et al. Changes in inflammatory bowel disease subtype during follow-up and over time in 44,302 patients. Scand J Gastroenterol 2019;54:55-63.
