# Supplementary material for: Statin use and risk of colorectal cancer in patients with inflammatory bowel disease
Source: eClinicalMedicine. 2023 Aug 24;63:102182. doi: 10.1016/j.eclinm.2023.102182 (PMC10474364; doi:10.1016/j.eclinm.2023.102182)
Supplement: R1 Statin CRC protocol [file mmc2.docx]

**Statistical Analysis Plan**

**Statin use and risk of colorectal cancer in patients with inflammatory bowel disease**

Jiangwei Sun, PhD

[Jiangwei.sun@ki.se](mailto:Jiangwei.sun@ki.se)

Jonas F. Ludvigsson, MD, PhD

[jonasludvigsson@yahoo.com](mailto:jonasludvigsson@yahoo.com)

Department of Medical Epidemiology and Biostatistics

Karolinska Institutet

Stockholm

Sweden

**MODIFICATION HISTOY:**

| Version(Date) | Authors | Action/modification |
| --- | --- | --- |
| V1 (2022-01-12) | Jiangwei Sun, Jonas F. Ludvigsson | Created this statistical analysis plan (SAP) |
| V2 (2022-09-28) | Jiangwei Sun, Jonas F. Ludvigsson | Revised SAP based on all coauthors' comments |
| V3 (2023-06-30) | Jiangwei Sun, Jonas F. Ludvigsson | Revised SAP based on comments from external reviewers |

**COAUTHORS:**

IBD experts: Jonas Halfvarson, Ola Olén

Statin expert: Paul Lochhead

Colorectal cancer expert: Mingyang Song

ESPRESSO team: David Bergman, Fahim Ebrahimi, Bjorn Roelstraete

**STUDY OBJECTIVES**

To investigate the role of statin use on the risk of colorectal cancer (CRC), CRC-related mortality, and all-cause mortality in patients with inflammatory bowel disease (IBD).

**HYPOTHESIS**

Statin use is associated with lower risk of the development of CRC, CRC-related mortality, and all-cause mortality in patients with IBD.

**METHOD**

**Data source, study design, and study population**

This cohort study will be based on the nationwide histopathology cohort, Epidemiology Strengthened by histoPathology Reports in Sweden (ESPRESSO)^1^, and several healthcare registers in Sweden. ESPRESSO^1^ contains data on GI-related biopsies from all 28 pathology departments in Sweden from 1965 to 2017. The Total Population Register^2^ contains details on birth, death, and migration, which was initiated in 1968. The Prescribed Drug Register^3^ collects information on medications dispensed at all pharmacies in Sweden since July 2005, including for example dispensing date, drug name, and a defined daily dose (DDD). The National Patient Register^4^ covers data on inpatient (since 1964) and outpatient (since 2001) specialist care with disease diagnoses and surgical procedures. The Cancer Register^5^ contains data from >96% of incident cancer cases since 1958. The Cause of Death Register^6^ contains information on >99% of death since 1952. The Swedish Longitudinal Integrated Database for Health Insurance and Labour Market Studies^7^ contains information on demographic and socioeconomic characteristics (e.g., education and county of residence).

The IBD patients will be identified as those with ≥ 1 biopsy indicating IBD in the ESPRESSO and ≥ 1 International Classification of Disease (ICD) code for IBD in the NPR (see **Table 1** for the definition of IBD subtypes)^8^. Such diagnostic approach yields a positive predictive value of 95%^9^.

**Identification of statin users**

Statin use will be identified from the Prescribed Drug Register by the Anatomical Therapeutic Chemical (ATC) code C10AA, including lipophilic statins (simvastatin and atorvastatin) and hydrophilic statins (pravastatin and rosuvastatin) (see **Table 2** for ATC codes). Statin users will be identified as those aged ≥ 18 years and with a statin prescription for ≥30 cumulative DDD (cDDD) from July, 2006 or later. The date that an individual first attained a cDDD ≥ 30 for a statin will be defined as the index date for statin exposure. Individuals who had any statin prescription before July 2006, before IBD diagnosis or before the first attainment of cDDD ≥ 30 will be excluded from the analysis to ensure a new-user design. We will use the intention-to-treat analysis as the main analysis.

**Two-step matching process**

A two-step matching process will be applied to establish the cohort. In the direct matching step, statin users and non-statin users will be matched on age at IBD diagnosis (<18, 18-<40, 40-<60, and ≥60), sex, IBD subtype (CD or UC), and calendar year at IBD diagnosis (1969-1989, 1990-1999, 2000-2009, 2010-2018). IBD-unclassified will not be considered in this project due to diagnostic uncertainty. To ensure similar opportunities for care and treatment^10^, non-statin users should have a random drug prescription (except IBD medications, see **Table 2** for ATC codes) within three months before or after the start date of statin treatment in the matched statin users. All identified non-statin users will be assigned the same index date as the index statin user. We will exclude individuals who had a record of migration, received a colectomy, or had a diagnosis of any of the following diseases before the index date: cancers, human immunodeficiency virus/ acquired immunodeficiency syndrome, primary immunodeficiency disease, end-stage renal disease, severe liver failure, and any organ transplantation (see **Table 2** for procedure and ICD codes).

In the propensity score matching step, statin users and non-statin users will be matched in a 1:1 ratio by using a greedy nearest-neighbor matching algorithm without replacement, with a caliper of 0.2 standard deviation of the logit of the propensity score^11^. A logistic regression model will be used to calculate the propensity score after adjusting for the below demographic and socioeconomic characteristics, comorbidities, and co-medications identified up to and including the index date.

- Age at IBD diagnosis, years (continuous);
- Duration of IBD, years (continuous);
- Number of healthcare visits (continuous);
- Country of birth (Nordic countries or others);
- Education (0-9, 10-12, ≥13 years, or missing);
- Comorbidities (yes/no, see Table 2 for detail): gastrointestinal diseases, intestinal infections, cardiovascular diseases, obesity/dyslipidemia, type 1 and type 2 diabetes, autoimmune diseases, obstructive sleep apnea, chronic obstructive pulmonary disease, and myositis;
- Medications (yes/no, see Table 2 for detail): antibiotics, proton pump inhibitor, antiviral medications, IBD medications, non-aspirin anti-platelet medications, aspirin, non-statin lipid lowering medications, anti-diabetic medications, anticoagulation medications, and anti-arrhythmias medications.

To examine if the matching has worked we will calculate standardized mean differences and comparing propensity score distribution and balance. A covariate with a standardized mean difference less than 10% will be considered well balanced.

**Identification of primary and secondary outcomes**

Follow-up will begin at index date until the first recorded date of incident CRC, proctocolectomy, emigration, death, or end of follow-up (Dec, 2019). A patient who had a colectomy but had an intact rectum will be considered at risk of rectal cancer until proctectomy (see **Table 2** for procedure codes). The primary outcome is incident CRC (see **Table 2** for ICD codes), while secondary outcomes are CRC-related mortality and all-cause mortality (see **Table 2** for ICD codes).

**STATISTICAL ANALYSES**

Incidence rates and their differences with 95% confidence intervals (CIs) for incident CRC, CRC-related mortality, and all-cause mortality will be calculated using Poisson regression. To account for competing risk, we will apply subdistribution hazard model to estimate hazard ratios (HRs) and 95% CIs, using time since index date as the underlying time scale. Cumulative incidence function will be applied to estimate the cumulative incidence of the primary and secondary outcomes.

We will calculate the number needed to treat (NNT)^12^ to avoid one event at 10-year since it is the most commonly used time period for CRC^13^.

**Secondary analyses**

Three secondary analyses will be conducted. First, to assess the relationship between continuous statin treatment and outcomes, we will apply an as-treated analysis where we further censor the follow-up at the end of the first treatment episode, in addition to the abovementioned censoring criteria. Second, to evaluate whether cumulative duration of statin use is associated with the outcomes, we will apply a nested case-control study in statin users. Using the method of incidence density sampling, for each case exposed to statin and with later studied outcomes, we will randomly select up to five statin users who did not develop the interested outcomes (controls). They will be individually matched on age at index date, gender, IBD subtype, IBD duration, and duration of follow-up. Conditional logistic regression will be used to estimate odds ratios (ORs) with 95% CIs. The cumulative duration of statin use will be assessed between first prescription and end of follow-up, and categorized into categories (30 days-<1 year (reference group), 1-<2 years, 2-<5 years, and ≥5 years cDDD). Trends in the dose-response association will be tested by using the duration strata as an independent variable in the model. Third, we will repeat the main analysis without accounting for competing risks.

**Subgroup analyses and sensitivity analyses**

We will assess whether the associations vary by the following factors: age at the index date (<60 or ≥60 years), sex, calendar period for IBD diagnosis (1969-1999 or 2000-2018), educational attainment (0-9, 10-12, ≥13 years, or “missing”), IBD subtype (CD or UC), age at IBD diagnosis (<50 or ≥50 years), duration of IBD (<10 or ≥10 years), type of statins initially treated, and type of CRC (colon cancer or rectal cancer).

Several sensitivity analyses will be conducted. First, we will construct a model in which covariates will adjusted as time-varying variables. Second, we will exclude individuals with a history of CVD before statin initiation. Third, we will discard the first year and then first two years of follow-up from the analysis. Fourth, we will evaluate the potential influence from unmeasured confounding by calculating E-values^14^. Finally, we will perform a negative control analysis using fracture as a negative control outcome to assess potential biases in our study^15^.

Data analyses will be performed using SAS version 9.4, Stata (version 16.1), and R version 3.6.0.

**Ethical approval**

This project will be conducted with ethical approvals from the Stockholm Ethics Review Board (2014/1287-31/4, 2018/972-32, and 2022-05774-02).

*Non-prespecified Analysis (Analysis according to comments from external reviewers):*

- Assess the association between statin use and risk of left-sided or right-sided colon cancer;
- Assess the association between statin use and cancer stage (measured by TNM classification and categorized into two groups: I-II or III-IV);
- To assess the robustness of our results, we added one sensitivity analysis by excluding individuals with a history of diabetes before statin initiation.

| Table 1. International Classification of Disease (ICD) codes and SNOMED codes for defining IBD | | | | | |
| --- | --- | --- | --- | --- | --- |
| IBD subtypes ^a^ | ICD-7 (1964-1968) | ICD-8 (1969-1986) | ICD-9 (1987-1996) | ICD-10 (1997-) | SNOMED codes ^b^ |
| Ulcerative colitis (UC) | 572,20; 572,21; 578,03 | 563,1; 563,10; 569,02; 569,04 | 556 | K51 | D6255 or M41, M42, M43, M44, M463, or M47 |
| Crohn's disease (CD) | 572,00; 572,09 | 563,00 | 555 | K50 | D6216 or M41, M42, M43, M44, M463, or M47 |
| IBD unclassified (IBD-U) | UC + CD | UC + CD or 563; 563,0; 563,9; 563,98; 563,99 | UC + CD | UC + CD or K52.3 | D6214 or M41, M42, M43, M44, M463, or M47 |
| ^a^ Subtypes of IBD were defined according to the first two diagnostic codes only, therefore no information after start of follow-up contributed to such definition; and the IBD subtype was only determined by the ICD code if one individual had one ICD code for IBD and one M code. ≥1 ICD code for IBD plus a relevant biopsy code has a positive predictive value of 95% ^9,16^. In a recent paper ^17^, we report that 18% of incident IBD patients in the Swedish patient register during 2002-2014 were classified as another IBD subtype at some point during follow-up. | | | | | |
| ^b^ SNOMED codes starting with M (inflammation suggestive of IBD, but not a specified subtype) were required to be accompanied by a topographic code of T67 or T68 (colon); for example, M41 refers to all codes starting with M41; D codes are diagnostic codes but listed under morphology in pathology registers; D6255, for example, is the diagnostic code for UC. | | | | | |

| Table 2. Definitions of statin, outcomes, exclusion criteria, comorbidities, and medications | |
| --- | --- |
| Statin | **ATC code** ^a^ |
| Simvastatin | C10AA01 |
| Pravastatin | C10AA03 |
| Atorvastatin | C10AA05 |
| Rosuvastatin | C10AA07 |
| **Outcomes** | **ICD code** ^a^ |
| Incident colorectal cancer (CRC) ^b^ | ICD-7: 153-154, but not 1541 (anal cancer) or 1534 (appendiceal cancer) |
| CRC-related mortality | ICD-10: C18-20, but not C181 (appendiceal cancer) |
| All-cause mortality | ICD-10: A00-U99 |
| **Proctocolectomy and colectomy** | **Procedure code** |
| Proctocolectomy ^c^ |  |
|  | Sixth version (1969-1996): 4652, 4653, 4654 |
|  | Seventh version (1997-): JFH20-JFH40 |
| Colectomy with an intact rectum ^d^ |  |
|  | Sixth version (1969-1996): 4650, 4651 |
|  | Seventh version (1997-): JFH00, JFH01, JFH10, JFH11, JFH96 |
| Rectum amputation after a colectomy with an intact rectum ^e^ |  |
|  | Sixth version (1969-1996): 4820, 4821, 4822, 4828 |
|  | Seventh version (1997-): JGB00-JGB97 |
| **Exclusion criteria** | **ICD-10 code/procedure code** |
| Cancer | C00-C97, except non-melanoma skin cancer (C44) |
| HIV/AIDS | B20-B24, F024, O987, R75, Z114, Z219, Z711 |
| Primary immunodeficiency disease | D71, D80–D84 |
| Severe liver failure | B150, B160, B162, B190, K704, K72, K766, I85 |
| End-stage renal disease | N18.5,  N18.6, Z49, Z99.2, Z94.0; Procedure codes: 9200; V9200; 9212; V9212; 9314; V9531; DR012; DR013; DR016; DR024; QF006; 9211; V9211; 9213; V9213; V9532; DR015; DR023; DR055; DV056; 9219; V9219; 9223; V9223; DR017; DR020; DR055; DR056; 6070; KAS10; KAS20 |
| Any organ transplantation | Swedish surgery codes (KVÅ codes): KAS, FQA, FQB, GDG, JJC |
| **Comorbidities** | **ICD-10 code** |
| Gastrointestinal diseases | K00-K99 |
| Intestinal infections | A00-A09 |
| Cardiovascular diseases | I00-I99 |
| Obesity/dyslipidemia | E78 , E65, E66 |
| Type 1 and type 2 diabetes | E10-E14 , O24 |
| Autoimmune diseases | E035, E039, E050, E055, E059, E063, E065, G35, M05-M09, M32, L40 |
| Obstructive sleep apnea | G473 |
| Chronic obstructive pulmonary disease (only if diagnosed ≥40 years) | J41-J44 |
| Myositis (no adequate codes present for rhabdomyolysis) | M60 |
| **Medications** | **ATC code** |
| Antibiotics | J01 |
| Proton pump inhibitor | A02BC |
| Antiviral drugs | J05 |
| IBD medications |  |
| Immunomodulators |  |
| Azathioprine | L04AX01 |
| Mercaptopurine | L01BB02 |
| Methotrexate | L04AX03/L01BA01 |
| Anti-TNF treatment |  |
| Infliximab | L04AB02 (L04AA12 before 2008) |
| Adalimumab | L04AB04 (L04AA17 before 2008) |
| Golimumab | L04AB06 |
| Vedolizumab | L04AA33 |
| Systemic corticosteroids |  |
| Betamethasone | H02AB01 |
| Dexamethasone | H02AB02 |
| Methylprednisolone | H02AB04 |
| Prednisolone | H02AB06 |
| Prednisone | H02AB07 |
| Hydrocortisone | H02AB09 |
| Cortisone | H02AB10 |
| Systemic aminosalicylates (5-ASA) |  |
| Sulfasalazine | A07EC01 |
| Mesalazine | A07EC02 |
| Olsalazine | A07EC03 |
| Balsalazide | A07EC04 |
| Rectal aminosalicylates (5-ASA) |  |
| Mesalazine | A07EC02 |
| Corticosteroids acting locally |  |
| Hydrocortisone | A07EA02 |
| Budesonide | A07EA06 |
| Non-aspirin anti-platelet medications | B01AC excluding aspirin (B01AC06) |
| Aspirin | B01AC06 |
| Non-statin lipid lowering medications | C10AB, C10AC, C10AD, C10AX01-14 |
| Anti-diabetic medications | A10 |
| Anticoagulation medications | B01AA, B01AE, B01AF, B01AX |
| Anti-arrhythmias medications | C01BA, C01BB, C01BC, C01BD, C01BG |
| ATC: Anatomical Therapeutic Chemical; CRC: colorectal cancer; HIV/AIDS: human immunodeficiency virus/ acquired immunodeficiency syndrome; ICD: the International Classification of Disease. | |
| ^a^ ATC codes will be identified from the Prescribed Drug Register; ICD codes and procedure codes will be identified from the National Patient Register. | |
| ^b^ Incident CRC will be ascertained from the Cancer Register, while CRC-related mortality will be ascertained from the Cause of Death Register. In the Cancer Register, all ICD-8, ICD-9, and ICD-10 codes were back-translated to ICD-7. | |
| ^c^ Not at risk of colorectal cancer. | |
| ^d^ Only at risk of rectal cancer. | |
| ^e^ Not at risk of colorectal cancer. | |

**Reference:**

1. Ludvigsson JF, Lashkariani M. Cohort profile: ESPRESSO (Epidemiology Strengthened by histoPathology Reports in Sweden). *Clin Epidemiol.* 2019;11:101-114.

2. Ludvigsson JF, Almqvist C, Bonamy AK, et al. Registers of the Swedish total population and their use in medical research. *Eur J Epidemiol.* 2016;31(2):125-136.

3. Wettermark B, Hammar N, Fored CM, et al. The new Swedish Prescribed Drug Register--opportunities for pharmacoepidemiological research and experience from the first six months. *Pharmacoepidemiol Drug Saf.* 2007;16(7):726-735.

4. Ludvigsson JF, Andersson E, Ekbom A, et al. External review and validation of the Swedish national inpatient register. *BMC Public Health.* 2011;11:450.

5. Barlow L, Westergren K, Holmberg L, Talback M. The completeness of the Swedish Cancer Register: a sample survey for year 1998. *Acta Oncol.* 2009;48(1):27-33.

6. Brooke HL, Talback M, Hornblad J, et al. The Swedish cause of death register. *Eur J Epidemiol.* 2017;32(9):765-773.

7. Ludvigsson JF, Svedberg P, Olen O, Bruze G, Neovius M. The longitudinal integrated database for health insurance and labour market studies (LISA) and its use in medical research. *Eur J Epidemiol.* 2019;34(4):423-437.

8. Forss A, Clements M, Bergman D, et al. A nationwide cohort study of the incidence of inflammatory bowel disease in Sweden from 1990 to 2014. *Aliment Pharmacol Ther.* 2022;55(6):691-699.

9. Nguyen LH, Örtqvist AK, Cao Y, et al. Antibiotic use and the development of inflammatory bowel disease: a national case-control study in Sweden. *The Lancet Gastroenterology & Hepatology.* 2020;5(11):986-995.

10. Simon TG, Duberg AS, Aleman S, et al. Lipophilic Statins and Risk for Hepatocellular Carcinoma and Death in Patients With Chronic Viral Hepatitis: Results From a Nationwide Swedish Population. *Annals of internal medicine.* 2019;171(5):318-327.

11. Austin PC. A comparison of 12 algorithms for matching on the propensity score. *Stat Med.* 2014;33(6):1057-1069.

12. Austin PC. Absolute risk reductions and numbers needed to treat can be obtained from adjusted survival models for time-to-event outcomes. *J Clin Epidemiol.* 2010;63(1):46-55.

13. Song M, Emilsson L, Bozorg SR, et al. Risk of colorectal cancer incidence and mortality after polypectomy: a Swedish record-linkage study. *The Lancet Gastroenterology & Hepatology.* 2020;5(6):537-547.

14. VanderWeele TJ, Ding P. Sensitivity Analysis in Observational Research: Introducing the E-Value. *Annals of internal medicine.* 2017;167(4):268-274.

15. Levintow SN, Orroth KK, Breskin A, et al. Use of negative control outcomes to assess the comparability of patients initiating lipid-lowering therapies. *Pharmacoepidemiol Drug Saf.* 2022;31(4):383-392.

16. Mouratidou N, Malmborg P, Jaras J, et al. Identification of Childhood-Onset Inflammatory Bowel Disease in Swedish Healthcare Registers: A Validation Study. *Clin Epidemiol.* 2022;14:591-600.

17. Everhov AH, Sachs MC, Malmborg P, et al. Changes in inflammatory bowel disease subtype during follow-up and over time in 44,302 patients. *Scand J Gastroenterol.* 2019;54(1):55-63.
